# Expanding the Prostate Cancer Cell Line Repertoire with ACRJ-PC28, an AR-negative Neuroendocrine Cell Line Derived From an African-Caribbean Patient

**DOI:** 10.1158/2767-9764.CRC-22-0245

**Published:** 2022-11-07

**Authors:** Henkel Valentine, William Aiken, Belinda Morrison, Ziran Zhao, Holly Fowle, Jason S. Wasserman, Elon Thompson, Warren Chin, Mark Young, Shannique Clarke, Denise Gibbs, Sharon Harrison, Wayne McLaughlin, Tim Kwok, Fang Jin, Kerry S. Campbell, Anelia Horvath, Rory Thompson, Norman H. Lee, Yan Zhou, Xavier Graña, Camille Ragin, Simone Badal

**Affiliations:** 1 Department of Basic Medical Sciences, Faculty of Medical Sciences Teaching and Research Complex, The University of the West Indies, Mona, Jamaica, West Indies.; 2 Department of Surgery, Radiology, Anaesthesia and Intensive Care, Section of Surgery, Faculty of Medical Sciences, The University of the West Indies, Mona, Jamaica.; 3 African-Caribbean Cancer Consortium, Philadelphia, Pennsylvania.; 4 Fels Institute for Cancer Research and Molecular Biology, Temple University Lewis Katz School of Medicine, Philadelphia, Pennsylvania.; 5 Department of Urology Kingston Public Hospital, North Street, Kingston.; 6 Cancer Prevention and Control Program, Fox Chase Cancer Center, Philadelphia, Pennsylvania.; 7 CARIGEN, Faculty of Medical Sciences Teaching and Research Complex, The University of the West Indies, Mona, Jamaica.; 8 Cell Culture Facility, Fox Chase Cancer Center, Philadelphia, Pennsylvania.; 9 Blood Cell Development and Function Program and Cell Culture Facility, Fox Chase Cancer Center, Philadelphia, Pennsylvania.; 10 Department of Biochemistry and Molecular Medicine, George Washington University School of Medicine and Health Sciences, Washington, District of Columbia.; 11 Department of Pathology, University Hospital of the West Indies, Mona, Kingston, Jamaica.; 12 Department of Pharmacology and Physiology, George Washington University School of Medicine and Health Sciences, GW Cancer Center, Washington, District of Columbia.; 13 Biostatistics and Bioinformatics Facility, Fox Chase Cancer Center, Philadelphia, Pennsylvania.

## Abstract

Prostate cancer cell lines from diverse backgrounds are important to addressing disparities in prostate cancer incidence and mortality rates among Black men. ACRJ-PC28 was developed from a transrectal needle biopsy and established via inactivation of the *CDKN2A* locus and simultaneous expression of human telomerase*.* Characterization assays included growth curve analysis, immunoblots, IHC, three-dimensional cultures, immunofluorescence imaging, confocal microscopy, flow cytometry, whole-genome sequencing (WGS), and RNA sequencing (RNA-seq). ACRJ-PC28 has been passaged more than 40 times *in vitro* over 10 months with a doubling time of 45 hours. Short tandem repeat profiling confirmed the novelty and human origin of the cell line. RNA-seq confirmed the expression of prostate specific genes alpha-methylacyl-CoA racemase (AMACR) and NKX3.1 and neuroendocrine specific markers synaptophysin and enolase 2 (ENO2) and IHC confirmed the presence of AMACR. Immunoblots indicated the cell line is of basal-luminal type; expresses p53 and pRB and is androgen receptor (AR) negative. WGS confirmed the absence of exonic mutations and the presence of intronic variants that appear to not affect function of AR, p53, and pRB. RNA-seq data revealed numerous *TP53* and *RB1* mRNA splice variants and the lack of *AR* mRNA expression. This is consistent with retention of p53 function in response to DNA damage and pRB function in response to contact inhibition. Soft agar anchorage-independent analysis indicated that the cells are transformed, confirmed by principal component analysis where ACRJ-PC28 cells cluster alongside other prostate cancer tumor tissues, yet was distinct. The novel methodology described should advance prostate cell line development, addressing the disparity in prostate cancer among Black men.

**Significance:**

Cell line development continues to attract less than 10% success rate. More than 98% of prostate cancer cell lines are from White men. This may contribute to the poorer response by Black men with prostate cancer to therapy compared with White men with prostate cancer, increasing overall survivorship among White men. The methodology described here to develop ACRJ-PC28, should advance the presence of Black prostate cancer cell lines thereby addressing prostate cancer disparity.

## Introduction

The use of *in vitro* cancer and normal models continues to be a yardstick for understanding the biological drivers of cancers with the goal of developing diagnostic, prognostic, and theragnostic tools. While *in vivo* models still provide more accurate predictions of human response, they are often used with human-derived two-dimensional (2D) models in the form of cell line–derived xenografts. Moreover, many factors support the complete transition to the application of *in vitro* models for cancer research; the protection of animals, more expedited and accurate identification of drug leads warranting clinical trials, and more cost effective and less labor-intensive approaches to anticancer research. Still, *in vitro* models have a long way to go before they can fully recapitulate *in vivo* models, whereby systems such as the immune response (1) and extracellular matrix (ECM; ref. 2) are factored in when developing tools for prostate cancer diagnosis and treatment. Technologies like organ on a chip (3) are a step in this direction, notwithstanding, the use of simple 2D models, namely cell lines, remain crucial to these. While there is an understanding that three-dimensional (3D) *in vitro* models better recapitulate the phenotype of cells in their original microenvironment (4), the cost of maintaining 3D models for long-term interrogation remains a limitation of their utility (5). Human-derived 2D models still remain relevant in basic science and are still the most common research model (6).

Prostate cancer has one of the greatest disparities in incidence and mortality rates (7–9) and we previously described (8) the possible reasons for this. What remains clear is the lack of equal representation of Blacks in *in vitro* models (10) and in clinical trials (11) when ascertaining the drivers of the disease and during the process of developing treatments. The rate of prostate cancer mortality is on the decline in the United States (12), but this is more evident among White men (13) while Black men in regions such as the Caribbean and Africa continue to experience elevated and increasing incidence and death from prostate cancer (14–16).

We believe that the underrepresentation of Black men at all stages of research, preclinical and clinical, is a contributing factor to current chemotherapy drugs being more effective in treating prostate cancer among White men (17), https://corporate.dukehealth.org/news-listing/new-non-hormonal-target-identified-advanced-prostate-cancer. Although the survival rate of prostate cancer among Black and White men is significantly improved when the disease is diagnosed early, Black men are known to still have poorer outcomes than their White counterparts (11). Designing therapies with more effective outcomes must consider a comprehensive assessment of patients, including their geographic locale and ethnic background. The use of cell lines in large numbers is believed to hold the key to predicting drug leads more accurately (18, 19). The absence of cell lines before now from regions where the incidence and mortality rates of prostate cancer are among the highest is unfavorable to addressing the disparity of prostate cancer among Black men in general, especially those from the Caribbean. We report the first cell line from the Caribbean, a region presenting with the second highest incidence and mortality rates of prostate cancer among Black men.

## Materials and Methods

### Sample Collection

Once the project obtained ethical approval (ECP, 91, 13/14) from the local institution board, Mona Campus Research Ethics Committee patients provided their written informed consent in accordance with the Declaration of Helsinki. We selected patients with PSA levels >4 ng/mL who were undergoing guided transrectal needle biopsy (TRNB) of the prostate. Also selected were patients with locally advanced or metastatic prostate cancer undergoing transurethral resection of the prostate (TURP) to relieve bladder outlet obstruction that corresponded to the inclusion criteria: males >18 years of age, Jamaican by birth or heritage of naturalization, patient diagnosed with prostate cancer, patients consenting to the study, and patients being followed at either The University Hospital of the West Indies or The Kingston Public Hospital. Patients who were diagnosed with a contagious illness, which might put the researcher at risk (e.g., HIV/AIDS), and those who were undergoing medical therapy known to affect serum PSA levels were excluded from the study. Samples were either collected from biopsy cores that were selected on the basis of abnormal PSA levels and/or palpable abnormality of the prostate suspicious for cancer on digital rectal examination or from TURP samples that were selected on the basis of a previously confirmed diagnosis of prostate cancer. All samples (TURP and TRNB) were collected and transported in RPMI (ATCC; #30-2001) supplemented with 5% FBS (ATCC; #30-2020), 1% penicillin/streptomycin (ATCC: #30-2300), and 0.1% amphotericin (ATCC; #30-2301) and processed within 3–4 hours of collection. Specifically, ACRJ-PC28 was developed from a TRNB sample. A total of 28 samples were collected over 5 years utilizing different culture conditions to realize ideal culture conditions for prostate cancer cells (Supplementary Table S1).

### Establishment of Primary Prostate Cancer Explants

Collected TRNB and TURP samples were washed in PBS to remove traces of blood and other contaminants after which RPMI with 10% FBS, 1% penicillin/streptomycin, and 0.1% amphotericin was added, and tissue sections diced into 1 mm^3^ fragments. Diced tissue fragments were added to a 6-well plate precoated with 3T3 STO fibroblast feeder cells containing DMEM: F12K (Thermo Fisher Scientific; #D6429 and #21127022) with 20% FBS and left to incubate for 3 days at 37°C and 5% CO_2_. Feeder cells were added to culture flasks 24 hours prior to sample collection. Medium was replaced every 2 days subsequently until prostate cancer cells were seen radiating from tissue chips after approximately 8–10 days. At this point, medium was replaced with Keratinocyte serum-free medium (KSFM, Thermo Fisher Scientific; #131-500A) supplemented with 5 ng/mL EGF (Millipore Sigma; #E9644), 50 μg/mL bovine pituitary extract (Millipore Sigma; #P1167), 100 ng/mL hydrocortisone (Millipore Sigma; #H0888), 5 μg/mL insulin (Millipore Sigma; #I9278), 5 μg/mL Apo-Transferin (Millipore Sigma; #T1147), 0.5 ng/mL recombinant TGFα (Millipore Sigma; #GF313), and 1 μmol/L Epinephrine (Millipore Sigma; #E4642).

### Transduction of Primary Prostate Cancer Cells with pLV-hTERT-IRES-hygro and lenticrisprv2-sg*CDKN2A*

Establishment of ACRJ-PC28 cells was accomplished through transduction with pLV-hTERT-IRES-hygro (Addgene; #85140) and lentiCRISPRv2-sg*CDKN2A*, as described previously (20). Briefly, primary prostate cancer cells at passage 4, were cotransduced with lentivirus conditioned medium of HEK-293T cells (RRID:CVCL_ZK70) transfected with pLV-hTERT-IRES-hygro (RRID:Addgene_85140), lentiCRISPRv2-sg*CDKN2A*, and packaging constructs using calcium phosphate. The lentivirus containing media was replaced with fresh lentiviral medium 24 hours after the initial transduction and two infections were done 24 hours apart. Cells were then washed with PBS and incubated with fresh media for 72 hours before addition of hygromycin (Millipore Sigma; #H3274, 25 μg/mL) and puromycin (Millipore Sigma; #P8833, 0.25 μg/mL (20).

### Authentication of ACRJ-PC28

A frozen vial of ACRJ-PC28 (3.0 × 10^6^ cells/mL) and a formalin-fixed, paraffin-embedded (FFPE) block of the original patient tumor were sent to IDEXX Bioanalytical to perform short tandem repeat (STR) profiling. This was done on two occasions (passage 4 and 6). Analysis of 16 different STR markers using PCR was conducted as well as *Mycoplasma* testing.

### Characterization of ACRJ-PC28 Using Growth Curve Analysis, Immunoblots, IHC, 3D Cultures, Immunofluorescence Imaging, and Confocal Microscopy

First, the doubling time was assessed by seeding ACRJ-PC28 at 5.0 × 10^4^ cells/mL into T-25 cell culture flasks and counting the cell population for an individual flask at 24-hour intervals over a 6-day period. ACRJ-PC28 cells were trypsinized and counted with Biolife Automated cell counter and results were used to plot a linear growth curve. Individual cell counts were used to assess doubling time using the formula: DT = [*t*(ln2)]/[ln(xf/xi)] where DT is doubling time, where xf is final cell count, xi is initial cell count, and *t* is the number of days. Next, Western blot analysis was performed to characterize the protein expression profile of ACRJ-PC28 (at passage 6) compared with well-characterized prostate cancer cell lines (PC-3, LNCaP, and DU-145) immortalized normal human prostate epithelial cells (hPrEC clone 1 and clone 2), and HEK-293T as described previously (20, 21).

3D spheroid culture was performed using ACRJ-PC28 at passage 6 as described previously (20). Growth factor–reduced Matrigel (Corning; REF 356231) was supplemented as described previously (22). The bottom layer of Matrigel was 40% while the top was 20%. Two hundred cells were seeded in each well between the Matrigel layers. Supplemented medium was added on top and replaced every other day for about 2 weeks. For immunofluorescence detection, spheroids were washed with PBS and fixed as described previously (20). Samples were incubated with primary antibodies in PBS-T for 60 minutes at room temperature or at 4°C overnight, washed with PBS three times, followed by fluorescence-conjugated secondary antibodies (Alexa Fluor 488 goat anti-rabbit IgG (H + L) (A-11034, RRID:AB_2576217), 488 donkey anti-rat IgG (H + L) (A-21208, RRID:AB_2535794), and/or 647 goat anti-mouse IgG (H + L) (A-21236, RRID:AB_2535805) were purchased from Invitrogen mixed with Phalloidin iFluor-555 (Abcam; #ab176756) for 60 minutes at room temperature. After rinsing three times with PBS, DAPI was applied to samples for 15 minutes. Samples were protected from light until imaging with a Leica TCS SP8 confocal microscope with 63x lens. Androgen receptor (AR) immunofluorescence of cells growing on glass slides was performed as described previously (21). Briefly, the cells were seeded on coverslips and cultured with medium containing DHT for 24 hours before fixation with 4% paraformaldehyde, followed by permeabilization with 0.1% Triton X-100 in PBS and blocking with 3% BSA. The cells were incubated with AR primary antibody for 1 hour at room temperature and secondary antibody as well as phalloidin for 45 minutes. Coverslips were mounted with mounting buffer containing DAPI to the slides and sealed with nail polish before imaging.

### Primary Antibodies

Anti-GAPDH (sc-47724), p16^INK4A^ (sc-468), p14^ARF^ (sc-8613), p63 (sc-25268, RRID:AB_628092), laminin β1 (sc-33709), integrin β1 (sc-59827), cyclin B1 (sc-245, RRID:AB_627338), and p27 (sc-528, RRID:AB_632129) were purchased from Santa Cruz Biotechnology. Anti-p53 (OP43-100UG, RRID:AB_213402) was purchased from Oncogene. Anti-Keratin 5 (#905503) and Keratin 18 (#628401) were purchased from BioLegend. Anti-CK18 (#4548), phospho-RB (#9313S), and Anti-AR (#5153S) were purchased from Cell Signaling Technology. Anti-Vimentin (ab92547) was from Abcam. Anti-E-Cadherin (610181, RRID:AB_397580) was from BD Biosciences.

### IHC

To compare the original tissue sample to the cell line and to further confirm that the cells are prostate in origin, several antibodies [AR, chromogranin A, synaptophysin, ER, E-cadherin, keratin 8–18, NKX3.1 (NK3 homebox), PSA, p16, p27, p53, p63, Prostate Cocktail (AMACR/p63/HMWCK), CD44, c-MYC, Ki67, and MUC1] were evaluated via IHC on the original TRNB prostatic cores and the derived ACRJ-PC28 cells at passage 7. The original TRNB cores were treated by submerging in 10% neutral buffered formalin for preservation after which the cores were processed, embedded in wax, and serially sectioned to a thickness of 4 μm then stained with hemotoxylin and eosin stain. After prostate cancer diagnosis, screening for the antibodies was outsourced to Vitro Molecular Laboratories. Tissue pretreatment was performed in either citrate pH 6-buffer or online (BondIII), followed by incubation with antibodies, polymer-based peroxidase detection (Novocastra), and DAB in an automated platform (Autostainer, Thermo Fisher Scientific or Bond III, Leica). Histopathologic analysis of the antibodies was done using a Nikon Eclipse Ci research microscope (Nikon Instruments Inc., Americas).

Given the immunophenotypic expression of the original patient sample, the ACRJ-PC28 cells were subjected to an IHC panel, aimed at possible clarifying cellular lineage, and identifying cellular markers indicative of malignancy. The stains utilized were nuclear and cytoplasmic prostatic epithelial cell markers (AR, NKX3.1, and PSA), indicators for neuroendocrine (NE) cell differentiation [chromogranin A and synaptophysin (SYP)], a marker of cellular proliferation (ki67), and a marker for basal cell expression (p63). In addition, surrogate markers for neoplastic transformation, that is, cellular adhesion and intranuclear dysregulation (CD44, c-MYC, and BCL-2) were also utilized. Cells were cultured until 80%–90% confluence was achieved to receive at least 10^7^ cells or more. Adherent cells were then collected and fixed in 10% formalin then resuspended in PBS at 4°C. Cells were immobilized in an agarose plug and transferred to a histology cassette for downstream processing and paraffin embedding, to facilitate sectioning of 3–5 μm FFPE tissue sections for histochemical (HC) and IHC testing using required HC stains, biochemical markers, or mAbs. The slides were sent to Vitro Molecular Laboratories for biomarker analysis mentioned prior in this section.

### Soft Agar Assay

Soft agar assay was used to assess the tumorigenicity of ACRJ-PC28 (23). A stock of 1.8% noble agar (Millipore Sigma, #A5421) was prepared in Hank's Balanced Salt Solution and autoclaved for sterilization. Bottom agar (0.6% noble agar) was prepared by mixing 1.8% stock solution with medium and then aliquoted into a 24-well plate to set for 10 minutes. Top agar (0.3% noble agar) was prepared by combining 1.8% noble agar with medium and ACRJ-PC28 cells at passage 7 (250, 1,000, or 5,000 cells). Top agar was solidified for 30 minutes after which 100 μL of medium with 10% FBS was added to each well. Media were replenished weekly, and colonies grown for 3 weeks after which they were fixed in methanol and visualized using 0.15% crystal violet.

### Contact Inhibition, Cell-cycle Analysis, and Response to Doxorubicin Treatment

On day 0, 150,000 cells were seeded in 6 cm plates and collected at the indicated times as cell density increased. The percentage of cells at different phases of the cell cycle was determined by measuring DNA content via propidium iodide (PI) staining followed by flow cytometric analysis (24). Briefly, cells were collected in 1% FBS in PBS, fixed by dropwise addition of 70% ethanol, and stored at 4°C for subsequent analysis. Fixed cells were washed and resuspended in 1% FBS in PBS followed by addition of PI and RNAse. DNA content was determined by flow cytometric analysis. Corresponding protein samples were assessed by Western blot analysis for protein markers. For p53 response to DNA damage, cells were treated with 1 μg/mL doxorubicin and collected at the indicated timepoints (20).

### DNA Extraction, Whole-genome Sequencing, RNA Sequencing, and Principal Component Analysis

DNA from ACRJ-PC28 at passage 5 was purified using a modified version of the DNeasy Blood and Tissue Kit (Qiagen; #69504) protocol and yield was determined using Nanodrop UV-VIS spectrophotometer. DNA sample was stored at −20°C prior to whole-genome sequencing (WGS) at a read depth of −50x that was outsourced at BGI Sequencing, Hong Kong. Sample concentration was determined using a fluorometer and microplate reader, and sample integrity and purity were determined using gel electrophoresis. WGS libraries were constructed from 31.6 ng of DNA after sample shearing, selection, end repair, and ligation to 3′ adenylated fragments. Ligated DNA was sized selected for lengths between 300 and 400 bp prior to circularization by splint oligo sequence (ssCir DNA) that were formatted to the final library. The library was qualified by Agilent Technologies 2100 bioanalyzer, amplified to more than 300 copies of one molecular in DNA nanoball, and sequenced by combinatorial Probe-Anchor Synthesis. Sequence reads were aligned to HG19 genome with Burrows-Wheeler Aligner (BWA; ref. 25) and GATK pipeline (26) was used for variant calling with local realignment around InDels after removing duplicates reads. The copy-number variants (CNV) were called using CNVnator v0.2.7 (27).

For RNA sequencing (RNA-seq), ACRJ-PC28 cells (passage 5) were trypsinized, washed once with PBS, and pelleted by centrifugation. The RNA was extracted with High Pure RNA Isolation Kit (Roche; #11 828 665 001). The RNA concentration was 1837.6 ng/μL and 260/280 ratio was 2.11. A paired-end library of the prostate cancer cell line was constructed using NEBNext Ultra Directional RNA kit and sequenced according to the Illumina protocol for the HiSeq2000 platform and sequenced to a targeted depth of >100 million paired-end reads (150 nt of length). Raw sequence data were processed using Illumina's RTA and CASAVA pipeline software, which include image analysis, base calling, sequence quality scoring, and quality control. Sequencing datasets were quality assessed using fastqc (v. 0.11.9). The sequencing reads were trimmed to remove adaptor-related sequences and reads with >3 consecutive bases of aggregate quality score <20 using Trimmomatic [v. 0.38 (27)]. All splice variant transcripts were then quantified using Salmon [v.1.2.0 (28)] in quasi-mapping mode, where the high-quality sequencing reads were aligned and quantified against the latest version of the human reference transcriptome assembly [Ensembl release 101, Homo_sapiens.GRCh38.cdna.all.fa (29)]. We define expressed splice variant as having a minimal expression value of 0.1 transcripts per million (TPM).

To conduct PCA, sequence reads of 50 The Cancer Genome Atlas Prostate Adenocarcinoma (TCGA PRAD) tumor and normal pair samples were aligned to human genome (hg38) using STAR (30). The number of raw counts in each known gene from the Ensembl database was enumerated using htseq-count from the HTSeq package (31). Differential expression between normal prostate and ACRJ-PC28 samples was assessed for statistical significance using the R/Bioconductor package DESeq2 (32). Genes with *P* value <0.01 and a fold change ≥1.5 were used for PCA including ACRJ-P28 RNA-seq data.

### Cytotoxic Response to Endemic Jamaican *Cannabis Sativa* Tetrahydrocannabinol Extract

The cytotoxicity of three tetrahydrocannabinol (THC) extracts (THC_EX1, THC_EX2, and THC_EX3) from the Jamaican *Cannabis sativa* plant was assessed using the MTS assay as described in G Malich, B Markovic, and C Winder (33) against novel ACRJ-PC28 cells and established PC-3 cells. ACRJ-PC28 cells and PC-3 cells were grown to 90% confluency, after which they were harvested and later reseeded at 20,000 cells per well in a 96-well plate followed by the addition of the extracts at concentrations 3–80 μg/mL for 24-hours. Absorbance readings were taken at 490 nm.

### Data Availability

RNA-seq expression and WGS data were uploaded in the Gene Expression Omnibus (GEO; GEO:GSE213223) and SRA (SAMN30844989) databases, respectively. Until the cell line, ACRJ-PC28 is available on the ATCC and ECACC platforms, it can be accessed upon request from the corresponding author.

## Results

**FIGURE 1:**
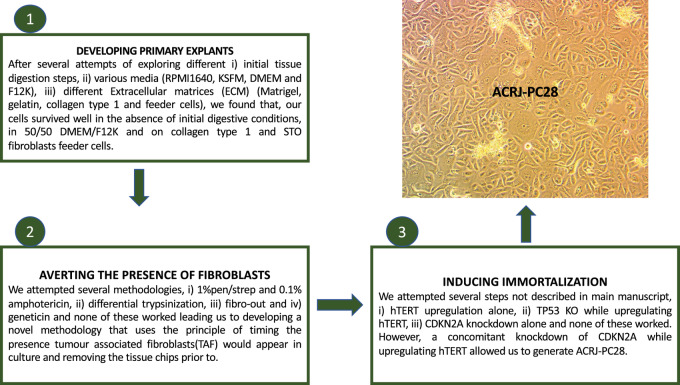
Steps utilized to generate ACRJ-PC28. The development of ACRJ-PC28 was primarily achieved by overcoming three challenges, (i) the identification of the appropriate media combination and ECM, (ii) overcoming the presence of TAF, and (iii) inducing immortalization using CDKN2A knockdown and hTERT upregulation.

We processed 28 prostate cancer samples over a 5-year period before successfully developing ACRJ-PC28 that was obtained from a 69-year-old Jamaican male with a PSA of 441.2 ng/mL, diagnosed with prostate cancer adenocarcinoma (Gleason score; 4+4) with perineural involvement. To confirm the ancestry of the cell line, admixture analysis using YRI and CEU from 1000 genome project was used as a reference, which indicated 97% African ancestry and male by birth. TRNB cores obtained from the patient's prostate were successfully grown to generate a primary prostate cancer explant which was then transduced with pLV-hTERT-IRES-hygro and lentiCRISPRv2-sg*CDKN2A* lentiviruses to establish an immortal ACRJ-PC28 cell line. We adapted numerous protocols (34–41) but experienced challenges to (i) develop primary explants (the first viably attached cells), (ii) avert the presence of fibroblasts, and (iii) attain either spontaneous or induced immortalization of the explants for their classification as cell lines, described in Fig. 1 Scheme 1. Discussions are underway to facilitate the inclusion of the cell line in ATCC's and ECACC's cell line repositories. In the meantime, persons interested in obtaining the cell line can contact the corresponding author.

### Generation of Primary Explants

**FIGURE 2:**
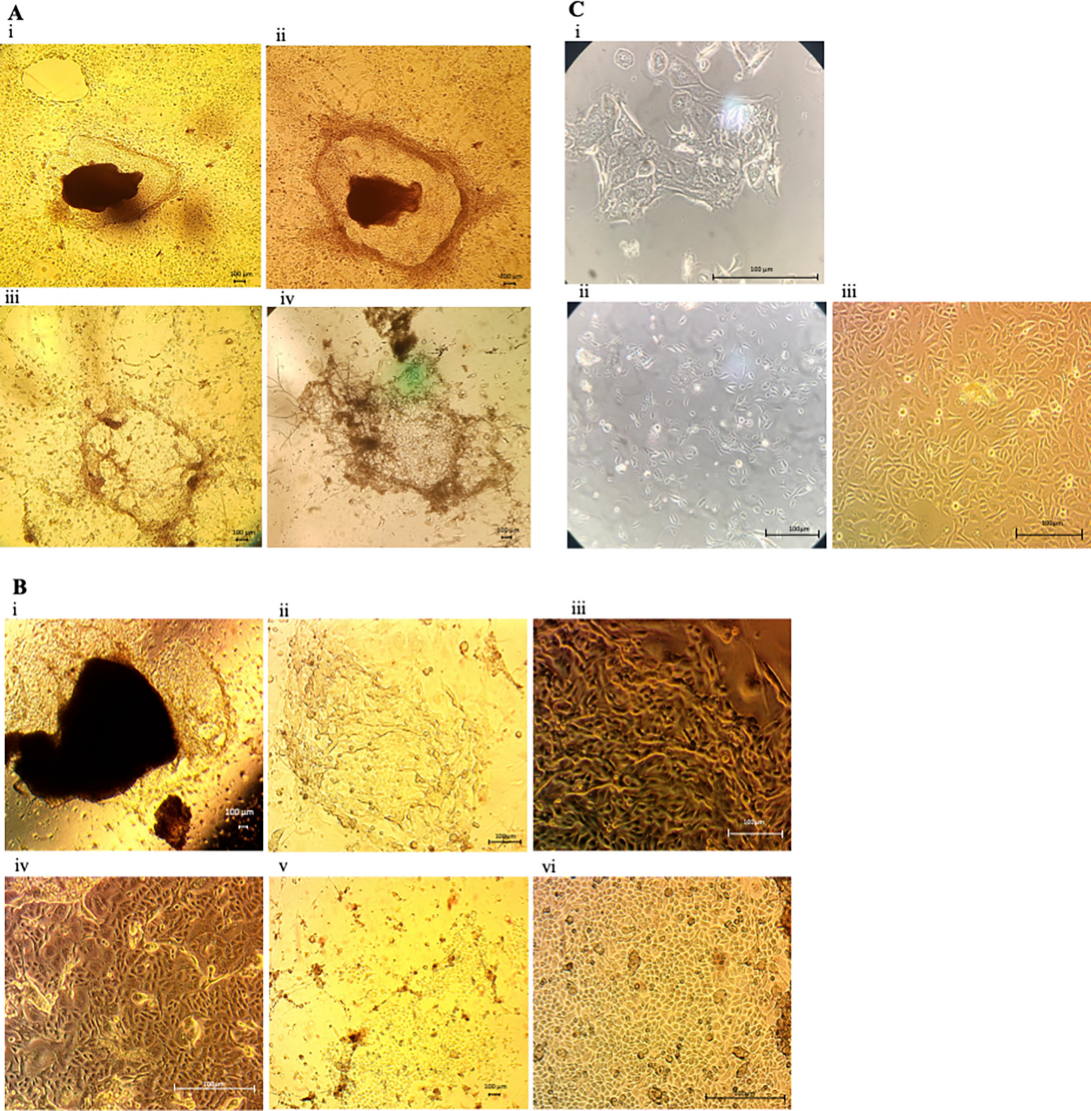
Stages involved in developing a prostate cancer cell line from primary explant (**A** and **B**) to immortalization (**C**). **A**, (i) Tissue fragment of ACRJ-PC13 attaches to Swiss 3T3 STO fibroblast feeder layer and begins to propagate radially from tissue fragment. (ii) Expansion of prostate cancer explant is restricted by intact 3T3 STO fibroblast feeder layer. (iii) Tissue fragments and fibroblast begin to detach with the addition of KSFM growth factor supplemented medium. (iv) Prostate cancer cells begin to expand beyond the border which existed due to 3T3 STO fibroblasts. **B**, (i) Tissue chip attached in DMEM: F12K medium with prostate cancer cells propagating radially from the tissue chip. (ii) Tissue chip was manually removed while prostate cancer cells continued to propagate. (iii) Propagating prostate cancer cells maintained consistent large cytoplasmic mass in 20% FBS. (iv) Decreased cytoplasmic mass in cells after reducing FBS to 10%. (v) ACRJ-PC17 following the change from DMEM:F12K to KSFM. (vi) Propagation of ACRJ-PC17 in KSFM. **C,** (i) ACRJ-PC28 cells after 10-day selection. (ii) Propagation of ACRJ-PC28 following successful transduction. (iii) ACRJ-PC28 at approximately 90% confluency.

Where mentioned in the literature, we recognized that the primary means of dissociating prostate cancer biospecimens applied in protocols (10) that describe the 32 currently established prostate cancer cell lines is that of enzymatic digestion followed by xenograft models. Attempting enzymatic dissociation with collagenase and hyaluronidase cocktails at various concentrations as detailed in Niranjan (34) and other protocols (35–41), gave us 0% success to develop our primary explants on the first 13 prostate cancer TURP samples. This led us to omit initial digestion steps from our protocols similar to Latimer (42) to keep prostate cancer fragments in an organoid-like form with the intention of mimicking *in situ* tumor microenvironment. Combining this step with the use of fibroblast feeder cells resulted in an almost 100% success rate in generating prostate primary explants. Interestingly, when we attempted to combine digestion steps with the use of the feeder cells, we observed no prostate primary explants. Perhaps, the use of digestion to generate primary explants for cell lines Bob, CA-HPV-10, MDA PCa 2b, RWPE-1, PZ-HPV-7, and HPE-15 was successful given the more resilient nature of the tumors they arose from. Nonetheless, as prostate cancer is widely heterogeneous, tumor resilience is expected to vary among samples and as such requires a methodology that is applicable across a myriad of tumours. DMEM: F12K with 20% FBS was identified as the optimal culture medium to promote attachment of prostate tissue fragments required to generate explants. Other media combinations used included, RPMI with 20% FBS, DMEM with 20% FBS, Ham's F12 with 20% FBS, and KSFM with EGF (5 ng/mL) and bovine pituitary extract (50 μg/mL), all of which were less efficient or completely ineffective (KSFM; Supplementary table S1). Using Swiss albino 3T3 irradiated feeder cells resulted in a 100% explant generation with approximately 50%–60% of attached tissue fragments that produced microexplants as shown in Fig. 2. Figure 2 also includes some of our successfully generated explants, ACRJ-PC13 and ACRJ-PC17, which did not transition into cell lines. Collagen type 1 was also an effective inducer of tissue fragment attachment, generating microexplants when used to coat culture vessels at 0.01%–0.02%, albeit less efficiently than Swiss albino 3T3 irradiated feeder cells with a 20%–30% attachment rate.

### Managing Tumor-associated Fibroblast Growth

The second hurdle we encountered was the presence of tumor-associated fibroblasts (TAF) that we observed at approximately 10 days or more among our primary explants. These TAF restricted the expansion of our explants through space (Fig. 2A) and resources. We observed that higher serum concentrations favored the viability of both our prostate primary explants and TAF. Decreasing the FBS concentration to 10% or below did reduce the presence of TAF but had negative effects on the morphology of our prostate primary explants. We observed a distorted morphology (Fig. 2B), and a 2-fold increase in cytoplasmic mass combined with earlier senescence (passage 3–4) compared with samples that were maintained in 15%–20% FBS that lasted up to the sixth passage. Next, we attempted mechanical removal of TAF, but this was ineffective as residual cells repopulated the culture. Differential trypsinization was also ineffective as prostate cells detached from flask around the same time as TAF. Other reagents, Human Prostate FibrOut (Chi Scientific; #7-15089) and gentamicin (ATCC, #30-230), resulted in death of primary prostate cells, while cocultured TAF remained unaffected. This led us to develop a novel methodology that gave us 100% success rate in averting the growth of TAF in our culture. First, we optimized the differential trypsinization protocol by recording the time to detach TAF in culture. Second, we recorded the most favorable (DMEM:F12K) and least favorable (KSFM) media for the growth of TAF and our prostate cancer epithelial cells. We then used DMEM:F12K to initiate prostate cancer epithelial cellular attachment and changed to KSFM in select subsequent cultures. Third, we recorded how long it took TAF to appear in culture (∼7–10 days) which was typically after our prostate primary explants (∼2–3 days). We then mechanically removed tissue chips at around day 5.

### Immortalizing Prostate-derived Explants—establishing ACRJ-PC28

The final hurdle was overcoming the eventual senescence of our cells. Of all the 28 samples we processed, none were spontaneously immortal, which is expected as spontaneous immortalization is a 1 in 10^7^ occurrence (43). A search within the catalogue of major cell line repositories ATCC and ECACC revealed that induced immortalization is common as where reported, around 44% of current prostate cancer cell lines were generated from induced immortalization (Supplementary table S2). The challenge we encountered was identifying a methodology that could result in little to no phenotypic changes from the original tumor sample. Our collaboration with Fox Chase Cancer Center (FCCC) and Temple University, developed a method by Zhao and colleagues (20), that is believed to provide a less transformative approach to immortalization compared with HPV-16/18 or SV-40 large-T/Small-t transduction methods given the normal features of the cell cycle are maintained when applying this method of immortalization to normal prostate epithelial cells.

What we routinely observed was that normally, our prostate extended explants senesced between passages 5–7. Described in Zhao and colleagues (20), was the principle that expression of p16^INK^ and p14^ARF^ from the *CDKN2A* locus and the lack of hTERT expression induce culture stress and replicative senescence, respectively. Thus, targeting both aids cells to bypass senescence. This proved successful in generating ACRJ-PC28 shown in Fig. 2C. *CDKN2A* ablation or hTERT upregulation individually proved insufficient to immortalize our prostate-derived explants. We also attempted the transduction of dominant-negative *TP53* coupled with hTERT expression, but this was also ineffective.

### Antibiotics Use is Associated with Earlier Onset of Senescence

We also sought to minimize the use of antibiotics in culture as numerous studies indicate that beta-lactam antibiotics, which include penicillin and aminoglycosides such as streptomycin, may affect gene expression and regulation, inducing antiproliferative effects in eukaryotic cells (44, 45). We observed that culturing prostate explants in media supplemented with penicillin and streptomycin experienced earlier onset of senescence, as early as passage 3 compared with samples grown without penicillin/streptomycin that senesced at around passages 5–7. As such, we adopted a model that limited the use of penicillin/streptomycin and amphotericin to only at the initial processing of tissue, after which all cells were grown in antibiotic- and antimycotic-free media.

### Authentication Profile of ACRJ-PC28

STR profiling (Supplementary Fig. S1) characterized ACRJ-PC28 as a novel human cell line by comparison with the DSMZ Human and Animal Cell Lines Database. *Mycoplasma* testing also proved that ACRJ-PC28 was negative for *Mycoplasma* contamination. We were unable to compare the original patient sample with the cell line as there was insufficient and fragmented DNA yield from the original FFPE block, which resulted in a lack of amplification of the human markers.

### Characterization of ACRJ-PC28

For WGS, using BWA, total clean reads aligned to human reference genome and on average 99.73% were mapped successfully and 88.13% were mapped uniquely. Overall, 4,184,160 SNPs were identified with 99.28% in dbSNP, 97.53% were annotated in the 1000 Genomes Project database, and 29,755 were novel. RNA-seq identified 79,687 expressed mRNA splice variants from a total of 16,675 genes annotated in the ENSEMBL genome database, for an average of 4.8 splice variants per gene. An in-depth analysis of the proteins encoded by the ACRJ-PC28 cell transcriptome was performed on the basis of the DAVID software (https://david.ncifcrf.gov). Supplementary Tables S3 and S4 summarize the findings of the WGS and RNA-seq data, respectively, that were used to further characterize and validate gene expression in the cell line, ACRJ-PC28.

### Immortalization Effects

**FIGURE 3:**
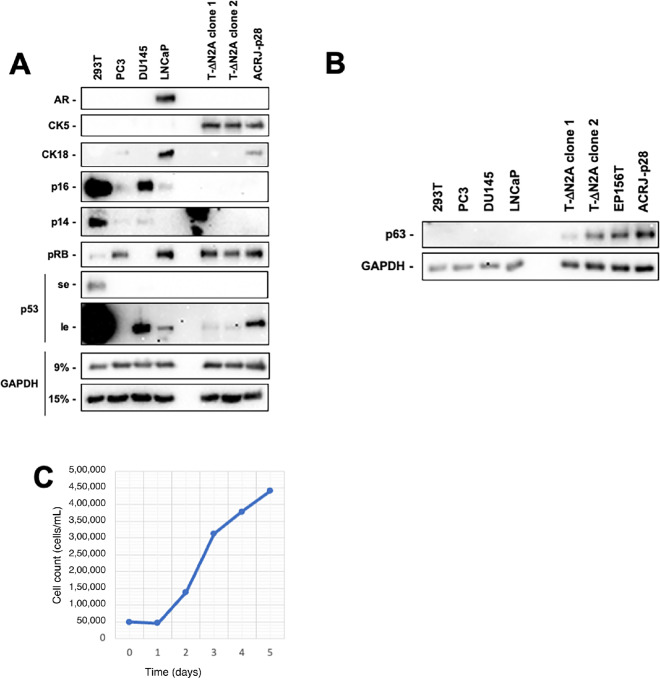
Characterization of immortalized human prostate cancer cell line ACRJ-PC28 in 2D. **A,** Expression analysis of AR, CK5, CK18, pRB, p53, p16, and p14 in ACRJ-PC28 and control cell lines (293T, PC3, DU145, LNCaP). ACRJ-PC28 cells express both CK5 and CK18. The absence of p16 and p14 expression confirming the successful knockout of CDKN2A. ACRJ-PC28 cells do not express detectable AR: B. LNCaP, DU145, and ACRJ-PC28 cells were seeded on coverslips and cultured with medium containing DHT for 24 hours before fixation. Cells were incubated with AR primary antibody followed by fluorescence conjugated secondary antibody and phalloidin. Coverslips were mounted with mounting buffer containing DAPI to the glass slides and sealed with nail polish before imaging with confocal microscope. Lens: 63×. Scale bar: 10 μm. Zoom factors are as indicated in the figure. Images confirm the absence of AR from ACRJ-PC28: C. The p63 basal marker is expressed in ACRJ-p28. cells. **B** and **C,** Protein expression levels were determined by Western blot analysis. **D,** Cells were counted at 1-day intervals over a 6-day period and indicates a doubling time of 45 hours.

Further characterization assays confirmed the absence of p16 and p14, downstream products of the *CDKN2A* gene that was knocked down (46) during the immortalization process. At the protein level, complete inactivation of p16 and p14 was achieved as ACRJ-PC28 (Fig. 3) had undetectable levels compared with positive controls DU-145, LNCaP, PC-3, and HEK293T (Fig. 3A). CNVnator indicated a possible heterozygous deletion at region chr9:21970801-22006400, which corresponds to the genomic region of exon 1 and exon 2 of *CDKN2A*. Interestingly, at the protein level, using IHC on the original patient sample, p16 expression and nuclear staining were limited to <1% of the cancer cells, with focal expression identified in some benign epithelial cells as well as stromal cells (Supplementary Fig. S2).

**FIGURE 4:**
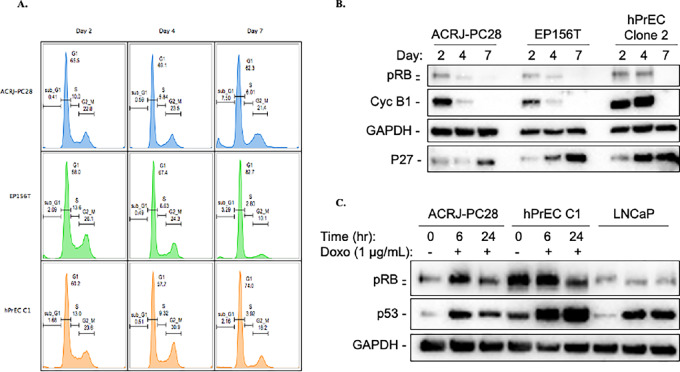
ACRJ-PC28 exhibit normal responses to contact inhibition cell-cycle arrest and chemotherapy induced DNA damage through functional pRB and p53 pathways. **A,** Growth to high cell density results in cell-cycle arrest. ACRJ-PC28, EP156T and hPrEC-C2 clone were allowed to grow to confluency. Cells were collected at the indicated times (in days) and cell-cycle arrest was determined by measuring DNA content by PI/flow cytometry analysis. **B,** G_0_ (quiescence) and mitotic markers were determined by Western blot analysis (relevant proteins are indicated). **C,** Cells were treated with 1 μmol/L doxorubicin for the indicated times (in hours) and processed for Western blot analysis (relevant proteins are indicated). Experiments shown are representative of three independent experiments.

Because pRB protein is readily expressed in ACRJ-PC28 cells, which was also confirmed using RNA-seq data, we assessed whether high cell density caused G_1_–G_0_ cell-cycle arrest (Fig. 4A), which is known to prompt activation of pRB and upregulation of p27 (47, 48). ACRJ-PC28 cells demonstrated sensitivity to increased cell-cell density comparable with immortalized normal hPRECs (EP156T and hPrEC clone 1), as evidenced by accumulations of cells with G_0_–G_1_ DNA content and a corresponding depletion of cells in S-G_2_–M at day 4 and most prominent at day 7 when cells reached full confluency (Fig. 4A). Consistent with these results, the levels of total pRB and mitotic cyclin B1 expression were depleted at day 4 and undetectable at day 7, while p27 expression peaked at day 7 (Fig. 4B). We also observed that when cells remained confluent for an extended period, cell death is detected (e.g.: hPrEC clone 1 in this experiment). Altogether, our results show that despite their cancerous origin, ACRJ-PC28 cells can be arrested by cell contacts when grown to confluency, which is consistent with their epithelial origin. This observation has been made for other cancer cell lines including T98G glioblastoma cells, which are transformed but demonstrate a normal pRB checkpoint (49, 50). Becaise ACRJ-PC28 cells express p53, we determined whether these cells exhibit a normal p53 response to genotoxic stress. As shown in Fig. 4C, treatment with the cytotoxic agent doxorubicin led to clear upregulation of p53 expression, although it was comparatively lower to the effect seen in normal immortal PrEC and LNCaP cells. The migration of the pRB band increased after 24 hours, which is indicative of dephosphorylation and associated with its activation downstream of p53. Therefore, ACRJ-PC28 cells retain at least partial p53 signaling.

IHC stains on the original tissue sample also revealed the presence of p27 that demonstrated a nuclear predominant staining pattern with a cytoplasmic blush, and this was seen in approximately 40% of the cancer cells; however, >80% of the benign ductal epithelial cells and basal cells also exhibited this staining pattern. p27 was also present in nuclei of stroma cells and stromal lymphocytes. p53 expression was limited to a solitary focus of prostatic tissue and within this focus, the cancer cells exhibited diffuse staining and the benign ductal epithelium demonstrated the usual wild-type staining pattern. Like the AR, WGS did not detect any exonic mutation in pRB and p53, whereas all intronic variants have no indication of affecting function based on all variant effect prediction tools (Supplementary Table S3).

### ACRJ-PC28 Cells are Transformed and Form Organoids

**FIGURE 5:**
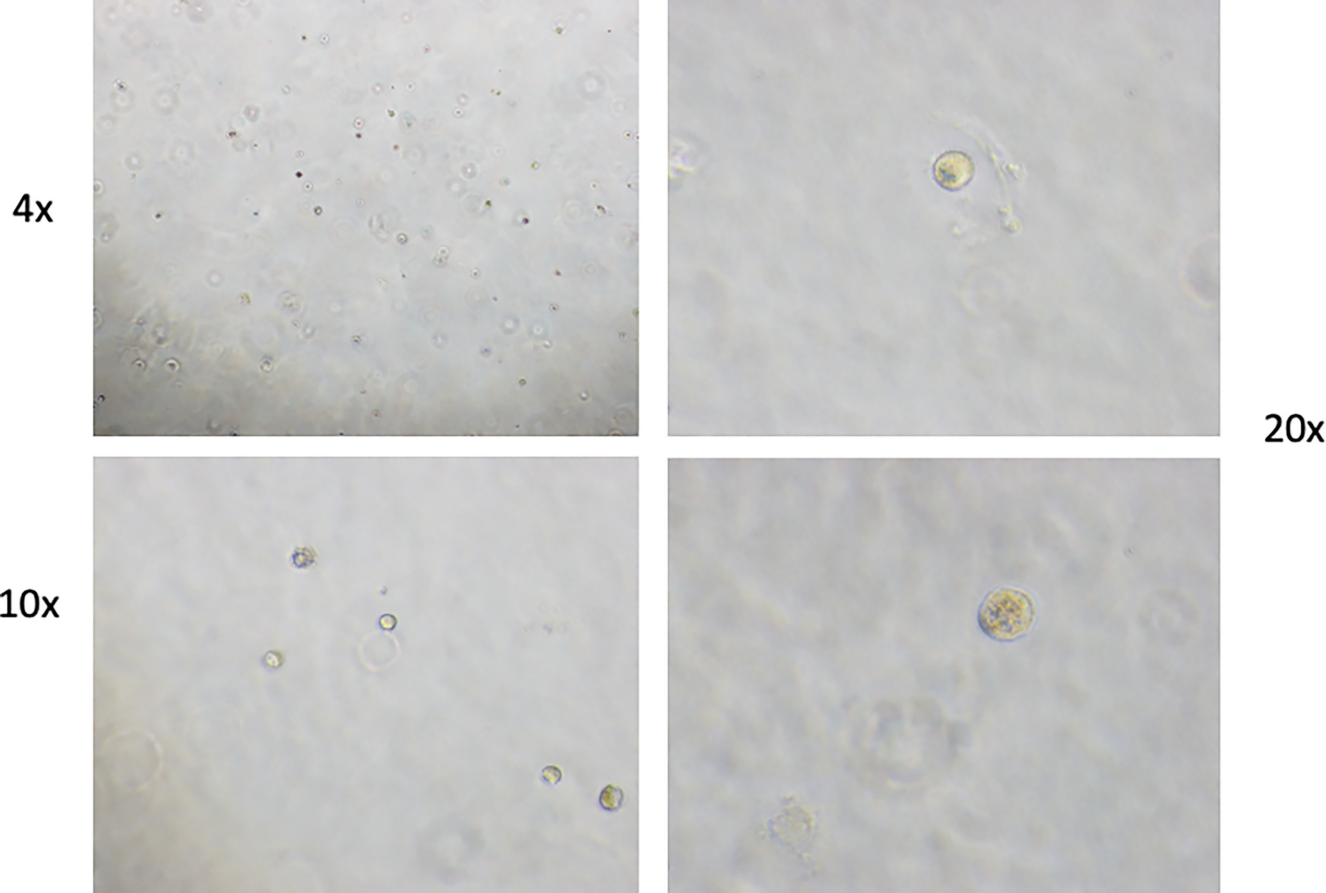
Soft agar assay to assess the tumorigenic potential of ACRJ-PC28. Cells were seeded at 250, 1,000, and 5,000 in 0.5 mL 0.3% soft agar and placed in a 24-well plate. We show here the plated 5,000 cells per well. The size of the colony is around 40 μm in diameter. Plating efficiency was roughly 23%.

**FIGURE 6:**
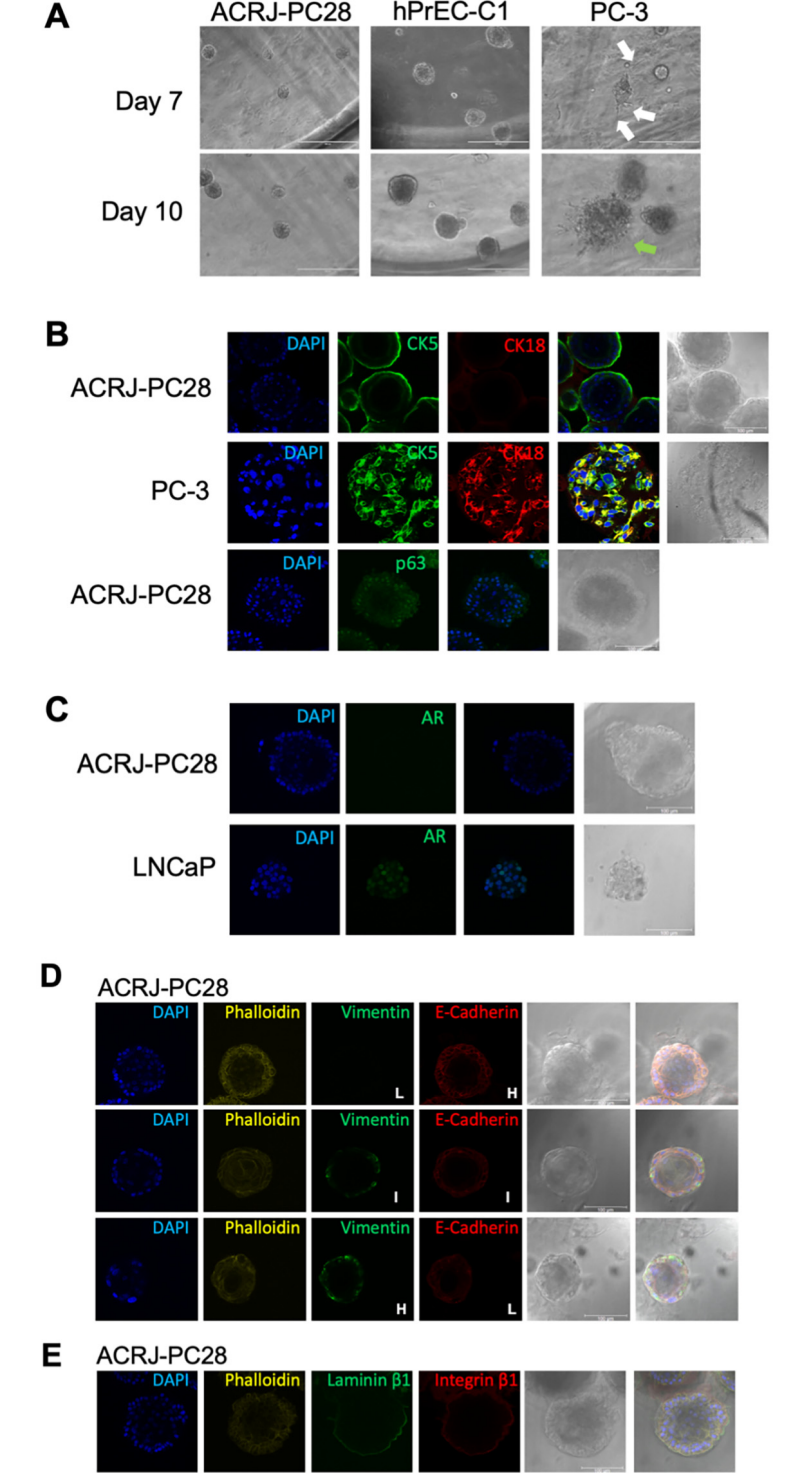
Characterization of immortalized human prostate cancer cell line ACRJ-PC28 growing as 3D organoids. **A,** ACRJ-PC28 cells were seeded on and embedded in Matrigel as described in the Materials and Methods and monitored over time by phase contrast microscopy. Organoids are shown at days 7 and 10. Lens 10×. Scale bar: 400 μm. Both ACRJ-PC28 and hPrEC-C1 cells form round organoids, but ACRJ-PC28 show lumens. PC-3 organoids form invasive protrusions (protrusions are marked with white arrows on day 7, and a green arrow marks the same organoid with many more protrusions). **B** and **C,** IF staining with CK5, CK18, and p63, and AR antibodies and imaged by confocal microscopy, 63×, demonstrates expression of basal markers CK5 and p63. However, expression of luminal marker CK18, and AR was negative. PC-3 and LNCaP organoids were used as positive controls for CK18 and AR, respectively. **D,** ACRJ-PC28 organoid vimentin and E-cadherin expression patterns suggest an opposite graded relative expression (designated H:high, I:intermediate, L:low). **E,** Expression of laminin β1 and integrin β1. **B**–**E,** Primary antibody incubation was followed by staining with fluorescence conjugated secondary antibody and phalloidin. Lens: 63×. Scale bar: 100 μm. Zoom factor: 0.75.

ACRJ-PC28 demonstrated successful anchorage-independent growth on soft agar, generating colonies at seeding densities of 5,000 cells per well in a 24-well plate as seen in Fig. 5. Lower densities of 250 and 1,000 cells per well failed to produce any surviving cell aggregates over the 3-week growth period. Plating efficiency was 23% with colonies growing to maximal size of 40 μm. Furthermore, ACRJ-PC28 organoids (Fig. 6) expressed protein expression of e-cadherin, integrin β1, and laminin β1 that were encased in the organoids seen in Fig. 6D. This was consistent with the original patient sample that also expressed E-cadherin (Table 1; Supplementary Fig. S2).

**TABLE 1:** Comparative Biomarker expression in original patient sample compared with ACRJ-PC28 and ACRJ-PC28 organoid: Biomarkers evaluated at the protein (IHC, Western blot analysis, and/or organoids) and/or RNA-seq in this research are listed here. Others mentioned in the discussion are not necessarily included in this table. Some biomarkers were not evaluated in either the original tissue, cell line, or organoids

		Methodology/Detection				
			ACRJ-PC28			
		Original tissue sample				ACRJ-PC28
(1)Biomarker	Biomarker specificity	IHC	IHC	WB	RNA-seq	Organoid
AMACR/P504	Prostate specific	✓	✓	−	✓	−
AR	Prostate specific	✓	x	x	x	x
NKX3.1	Prostate specific	✓	x	−	✓	−
PSA	Prostate specific	✓	x	−	x	−
PSCA	Prostate associated	−	−	−	✓	−
SLC45A3	Prostate associated	−	−	−	✓	
P63	Basal	✓/x	✓	✓	✓	✓
HMWCK	Basal	✓/x	−	−	✓	−
CK5	Basal	✓	−	✓	✓	✓
CK8-18	Basal & Luminal	✓	−	−	✓	−
CK18	Luminal	−	−	✓	✓	x
E-cadherin	Cell-cell binding	✓	−	−	✓	✓
Vimentin	Connective tissue	−	−		✓	✓
Integrin β1	Cell surface receptor	−	−	−	✓	✓
Laminin β1	Extracellular matrix	−	−	−	✓	✓
P16	Tumor suppressor	∼	−	x	x	−
P27	Tumor suppressor	✓	−	✓	✓	−
P53	Tumor suppressor	✓	−	✓	✓	−
PRB	Tumor suppressor	^	^	✓	✓	−
P14	Cell cycle regulator	^	^	x	x	−
CHGA	Neuroendocrine	✓	x	−	x	−
SYP	Neuroendocrine	x	x	−	✓	−
ENO2	Neuroendocrine	^	^	−	✓	−
MK164/K167	Cellular proliferation	✓	✓	−	✓	−
CD44	Immune receptor	✓	✓	−	✓	−
MYC	Oncogene	✓	✓	−	✓	−
KLF4	Cellular regulation	^	^	−	✓	−
MUC1*	Epithelial membrane	✓	✓	−	✓	−
POU5F1*	Germ cell regulation	^	^	−	✓	−
EZH2*	Maintains for embryonic development	^	^	−	✓	−
SOX2*	Transcription factor for stem cell maintenance	^	^	−	✓	−

NOTE: ✓, expressed; x, not expressed; –, not done; *, upregulated in tumors; ✓/x, present in basal cell/absent in luminal; ^I, HC biomarker available; ∼, equivocal.

Doubling time assessments demonstrated that ACRJ-PC28 had a doubling time of 45 hours as seen in Fig. 3C, which was slower than popular prostate cancer cell lines, PC-3 (∼29 hours) and DU-145 (∼34 hours), but as fast as LNCaP (∼28–60 hours), while being almost twice as fast as that of popular African American cell line MDA PCa 2b (∼80 hours).

### As Prostate Cancer

To confirm that ACRJ-PC28 is a prostate cancer cell line, we first examined at the protein level, the presence of prostate specific biomarkers, AR, AMACR, NKX3.1, and PSA (Table 1). AR levels were undetectable using western blot (Fig. 2A) and immunofluorescence (IF) staining (Fig. 6B). The formation of ACRJ-PC28 organoids in Matrigel and expression markers as described previously was also used to determine the presence of AR. ACRJ-PC28 organoids were not invasive as compared with PC-3 organoids that started to develop protrusions by days 6–7 (Fig. 6A). In agreement with ACRJ-PC28 cells grown in 2D culture, AR expression was not detected (Fig. 6B). The remaining biomarkers (AMACR, NKX3.1, and PSA) are routinely used in IHC analysis and so were evaluated in the original patient sample and ACRJ-PC28 (Table 1). IHC confirmed the presence of an invasive prostatic acinar adenocarcinoma with expression of alpha-methylacyl-CoA racemase (AMACR) in the cancer cells of the original patient sample. The cancer cells expressed strong diffuse nuclear expression for AR and NKX3.1. The PSA stain was also diffusely expressed; being present in the cytoplasm of the PSA-positive cancer cells. The NKX3.1 was strongly expressed only in the cancer cells whilst the non-neoplastic benign glandular epithelium exhibited patchy weak expression. AR expression was seen in varied intensities from low to high throughout the biopsy, as the stromal and benign tissues also demonstrated uptake of the immunostain. The cell line demonstrated strong nuclear expression for AMACR in 100% of the cells; however, there was no protein expression for AR, NKX3.1, and PSA in ACRJ-PC28.

We detected the prostate-specific biomarker AMACR in ACRJ-PC28 at the protein level even though all the other prostate-specific biomarkers (AR, NKX3.1, and PSA) were confirmed in the original patient sample. We then examined the WGS and RNA-seq data on the cell line. For AR, WGS did not detect any exonic mutation (Supplementary Table S3), while all intronic variants have unknown effects based on current variant effect prediction tools. Similar observations were obtained for AMACR, NKX3.1, and PSA. The absence of *AR* and *PSA* (gene symbol *KLK3*) expression was observed at the level of RNA-seq. However, *AMACR* and *NKX3.1* were expressed at the RNA level with TPM values of 1 or greater, but only AMACR was detected at the protein level. Furthermore, two prostate-associated markers, PSCA and SLC45A3, were also detected by RNA-seq with TPM values of 10 and 1, respectively.

**FIGURE 7:**
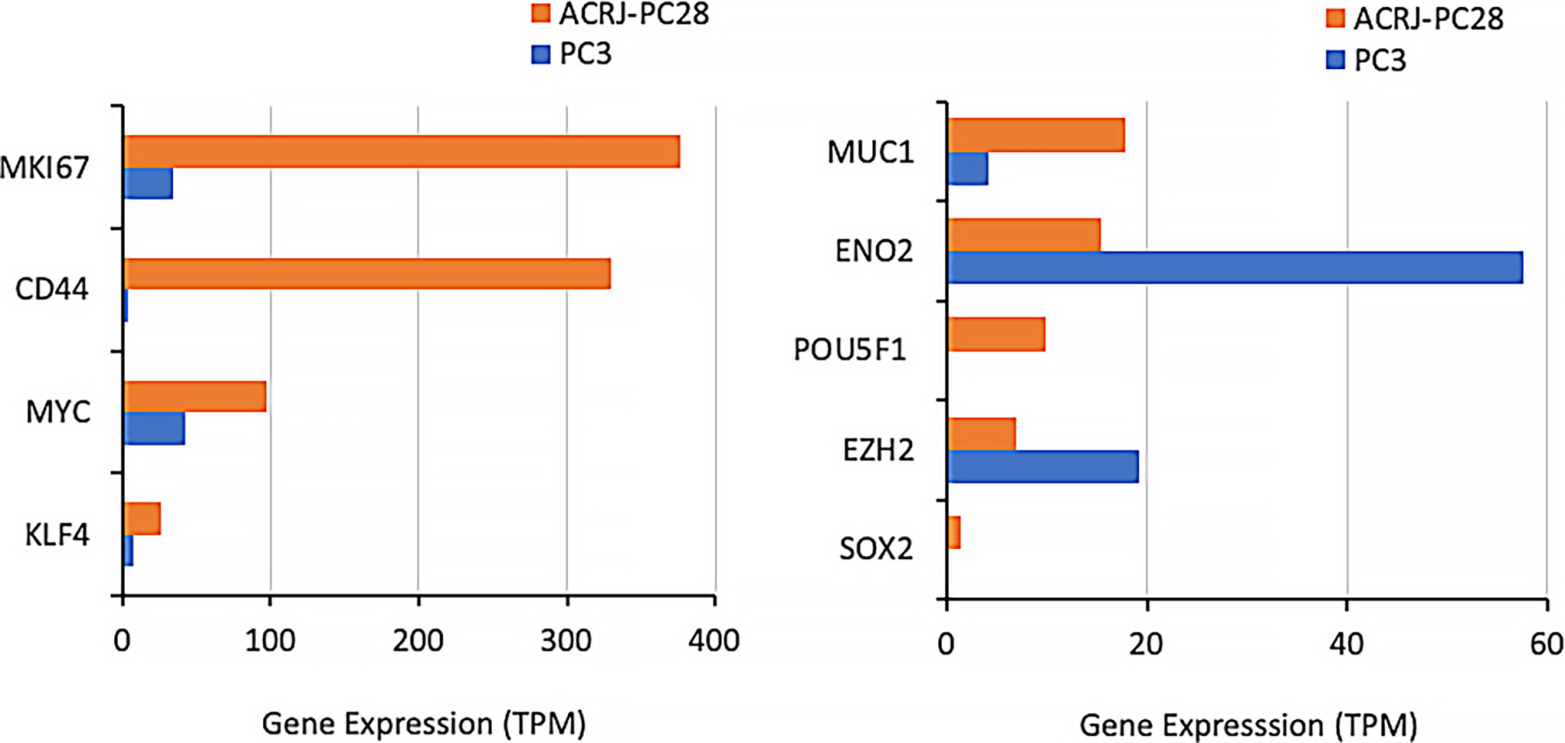
Seven of nine neuroendocrine markers are more highly expressed in ACRJ-PC28 compared with PC3.

It is common for prostate cancer with a NE phenotype to not express prostate cancer–specific biomarkers, AR, NKX3.1, and PSA (51). Therefore, we further characterized ACRJ-PC28 to evaluate the presence of commonly used NE markers, chromogranin A, SYP, and ENO2 using IHC on both the original patient sample and on the cell line. For the original sample, NE marker expression was limited to approximately 10% of the cancer cells and <5% of the benign ductal epithelium that demonstrated cytoplasmic positivity for chromogranin A stain. The SYP stain was negative in all neoplastic and non-neoplastic cells. There was no immunoexpression for chromogranin A and SYP in ACRJ-PC28, which agreed with the RNA-seq data for both biomarkers with TPM values <1. In contrast, RNA-seq indicated robust expression of *ENO2* with a TPM value of 10. We then evaluated the presence of several other genes that are not only upregulated in prostate cancer (Supplementary Table S5) but their overexpression has been associated with the prostate cancer NE phenotype. Seven of nine genes that are expressed in PC-3 cells [considered to have a NE-like phenotype (52)] were more highly expressed in ACRJ-PC28 (Fig. 7). Despite seven of nine expressed NE genes, the absence of the benchmark NE markers such as chromogranin and SYP begs for further interrogation into the possible NE phenotype of ACRJ-PC28 and validates the need for diverse prostate cancer cell lines. IHC confirms that the cell line demonstrated strong diffuse nuclear expression for CD44 and c-MYC in 100% of the cells and approximately 50% of the cells expressed MUC1. The Ki67 indicated proliferative activity in roughly 20% of the ACRJ-PC28.

**FIGURE 8:**
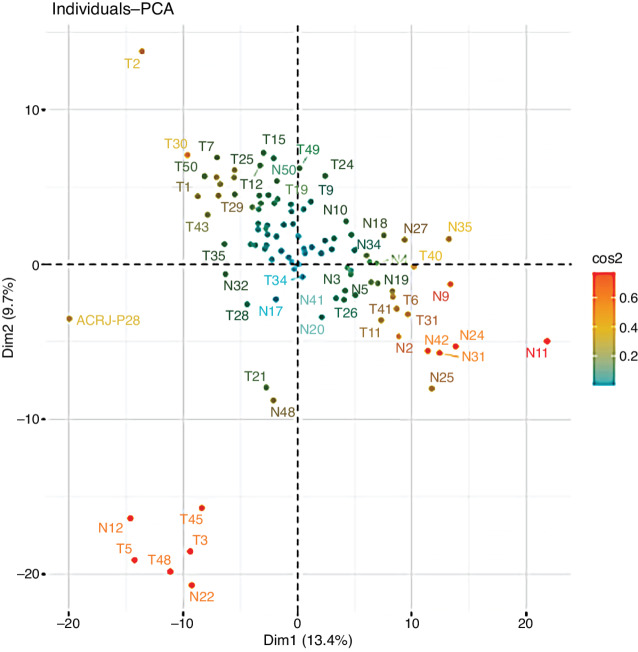
PCA plot of mRNA data of ACRJ-PC28 and 50 TCGA prostate Tumor (T) and normal (N) paired samples. Each dot represents a sample.

Next, we conducted a PCA using the RNA-seq data of 50 TCGA prostate normal and tumor paired samples and ACRJ-PC28. The analysis was adjusted by race and Gleason score. We used the top 169 genes that varied the most between normal and tumor samples. Results as seen in Fig. 8 showed that ACRJ-PC28 closely clustered with tumor samples from TCGA PRAD rather than normal samples. Interestingly, even though ACRJ-PC28 was among the tumor samples, it stood alone indicating the uniqueness in the cell line's genetic profile. Using the WGS data, we assessed the presence of variants and/or mutations in several genes that are known to be frequently mutated in prostate cancer (*BRCA1, BRCA2, RB, PTEN*, *CDK12, PI3K,* ADP-ribose, *PIK3GA, PIK3CB* and *PIK3R1*, *AKT, CYP17, IGF 1, EGF,* and *BCL2*; Supplementary Table S3). Five genes (*EGF, PIK3R1, ATM, BRCA1,* and *BRCA2*) appeared to have damaging mutations based on variant effect prediction and among the 5, *ATM* has a rare mutation (rs147934285) given the <1% population frequency. At the level of RNA-seq, there was a lack of expression of *EGF*, *BRCA1,* and *BRCA2* (TPM <1), whereas splice variant(s) of *PIK3R1* and *ATM* were detected. RNA-seq detected a very weak signal for one variant of *TMPRSS2* (TPM = 0.5).

Other biomarkers that distinguish basal (normal) and luminal (cancer) cells were evaluated in the original patient sample and the cell line. Using Western blot analysis, 2D cultures of ACRJ-PC28 were positive for basal markers (CK5 and p63) and a luminal marker (CK18; Fig. 3A and B), indicating that ACRJ-PC28 represents a possible basal-luminal transitional cell line. Meanwhile, ACRJ-PC28 organoids expressed both CK5 and p63, but surprisingly did not express CK18 when grown as 3D cultures (Fig. 6B and C). IHC on the original patient sample indicated that a CK8-18 cocktail was strongly expressed in all the cancer cells and benign epithelial cells, as well as all the basal cells. The p63 stain was however positive in the basal cells only. The cell line demonstrated strong diffuse nuclear expression for p63 in 100% of the cells.

### ACRJ-PC28 Bioactivity: Cytotoxic Response to Endemic Jamaican *Cannabis Sativa* THC Extract

Of the three THC extracts (THC_EX1, THC_EX2, and THC_EX3) assessed, one (THC_EX3) demonstrated noteworthy bioactivity (Supplementary Fig. S4). This extract was roughly three times more potent in reducing the viability of ACRJ-PC28 cells given the estimated IC_50_ <3.7 μg/mL, while against PC-3, the estimated IC_50_ was 1.2 μg/mL.

## Discussion

We believe the methodology described here should increase the success rate of prostate cell line development from biospecimens obtained from either TURP, TRNB, or radical prostatectomy surgeries. We report >90% success rate in generating primary explants that survived up to passage 7, and the immortalization protocol described in Zhao and colleagues (20) is believed to provide a reduced transformative approach to prostate cell line development. This is especially important considering the prior absence of cell lines from the Caribbean, furthermore, the only 2 men of African ancestry (MAA) prostate cancer cell lines commercially available for research are from the United States. Importantly, we developed a novel methodology to avert the presence of TAF in addition to confirming the potential negative effects of penicillin/streptomycin on primary prostate-derived cells. Together, these could be limiting steps to advancing the catalogue of prostate cancer cell lines. TAF outgrowth in human primary cultures has been well documented with several suggested methodologies for fibroblast exclusion (53, 54). These published methodologies however failed to exclude TAFs from our prostate cancer primary explants. Considering this, our novel method could be further tested with other tissue types, increasing the complement of tools for generation of primary cell lines from multiple tissue types. A major contributor to the negative growth effects observed with penicillin/streptomycin use could be related to the connectivity between penicillin/streptomycin and *activating transcription factor 3 (ATF3)*. *AFT3* functions as a stress response mediator and is involved in multiple oncogenic pathways including AR signaling that controls prostate cell proliferation (55). Studies have reported an upregulation in the differentially expressed gene *ATF3* in penicillin/streptomycin conditions (56). Considering our observations and evidence in literature, this further emphasizes the importance of omitting penicillin/streptomycin from primary cell culture protocols while optimizing culture conditions by using aseptic techniques in a sterile work area along with sterile reagents and media (57).

Ideally, the patient sample used to develop a cell line should also be used for STR profiling to confirm the source of the cell line in addition to being used for pathology reports and IHC staining. This model, however, seems more feasible when collecting tumor specimen from radical prostatectomy surgeries which could provide >5 g of tissue but would require a pathologist on-site to confirm the sample used for developing the cell line is the same for STR profile and IHC stains. This set-up continues to be a challenge for us at The UWI, Mona and for the duration of this project, we did not access samples from radical prostatectomies. This left us with TURPs and biopsies and for both, the lack of sufficient tissue sample persists because cell line protocols vary among patient samples requiring careful planning to ensure that the appropriate ECM/s, media, growth factors, and incubation times are employed. These can easily exhaust tissue samples which could otherwise be used for authentication and characterization purposes. Moreover, biopsied samples, the type used to generate ACRJ-PC28 yield relatively less tissue (200 mg vs. 1–2 g from TURPs) and because 12 cores are usually taken, it is likely that the sample used for IHC staining on the original patient sample is different from the core used to develop the cell line. It is important to address these so that a wider cross-section of prostate cancer cell lines can be generated, not only increasing the representation among races but prostate cancer grades as well. Perhaps, optimizing protocols for DNA extraction from FFPE is one route to addressing these. Despite our attempts, DNA content was either insufficient or highly fragmented resulting in our inability to confirm the source of ACRJ-PC28.

Not having confirmation that ACRJ-PC28 was from the original patient sample that definitively displayed tumor throughout, was the first challenge to appropriately demarcate the cell line as prostate cancer. The second was the lack of expression of AR at both the protein and RNA-seq levels. This led us to explore other prostate-specific biomarkers AMACR, NKX3.1 and PSA, and AMACR was expressed at the protein level while *AMACR* and *NKX3.1* were expressed at the RNA-seq level. Interestingly, both AMACR and NKX3.1 were expressed in the original tissue sample, and it is possible that the biopsied core used to generate the cell line is different from the one used for IHC analysis. However, if the cell line was developed from the same core analyzed, the lack of expression of NKX3.1 at the protein level could be due to protein degradation. In any event, the factors surrounding the relationship between mRNA and protein expression remain to be fully elucidated (58). The further RNA-seq expression of prostate related genes, *PSCA* and *SLC45A3,* are sufficient to deem ACRJ-PC28 a prostate-derived cell line, especially because NKX3.1 was the only prostate-specific biomarker detected in the Bob prostate cancer cell line (35).

The lack of expression of the prostate-specific genes (*AR*, *NKX3.1,* and *PSA*) at the protein level prompted the investigation of a possible NE prostate cancer phenotype, which was confirmed at the RNA-seq level when 2 NE specific marker (SYP and ENO2) were expressed at RNA-seq. Specific analysis of mRNA expression in normal prostate tissue revealed expression of both ENO2 and SYP albeit at low levels; however, no expression was observed for both following IHC staining (59) similar to our observation for ACRJ-PC28. The mechanism behind this incongruity remains to be elucidated. Furthermore, the overexpression of seven of nine NE-associated markers seen in ACRJ-PC28 compared with the PC-3 cell line further supports an NE phenotype of ACRJ-PC28 (52). It is common for a NE prostate cancer phenotype to not express AR and PSA and common for amplification of the genes aurora kinase A (*AURKA*) and N-myc (*MYCN* (51), both of which were overexpressed in ACRJ-PC28 at RNA-seq level and MYC was detected at the protein level. It is also possible that ACRJ-PC28 represents a prostate cancer NE phenotype as opposed to a normal NE phenotype as described by Yan and colleagues (60). This was addressed in four ways. First, a normal NE phenotype usually stains positive for basal markers like CK5 (61), while a prostate cancer NE usually stains for luminal markers like CK18 (62). Interestingly, ACRJ-PC28 expressed basal (p63 and CK5) and luminal (CK18) markers in 2D culture, confirmed by both immunoblots and IHC stain. The lack of expression of the CK18 marker when assessed in 3D models is not surprising as the differential expression of proteins among 2D and 3D models is established (63). Furthermore, 3D models are believed to recapitulate more *in vivo* conditions. This possibly means that ACRJ-PC28 represents a transitional prostate cancer cell line. Second, the aggressive proliferative nature of the prostate cells (64) is a determinant in differentiating between normal NE and prostate cancer NE phenotype. A prostate cancer NE tends to exhibit more aggressive growth rates relative to normal NE prostate cells. ACRJ-PC28 exhibited a similar doubling time to LNCaP which exhibits NE transdifferentiation *in vitro* (65). Furthermore, the tumorigenicity of ACRJ-PC28 was determined by an anchorage-independent growth assay, which confirmed that the cells are transformed. Indeed, our ongoing characterization of the cell line will provide further insight, especially through *in vivo* studies. Third, a normal NE prostate phenotype typically does not express BCL-2 (66) and ACRJ-PC28 demonstrated overexpression of BCL-2. Furthermore, inhibitors of BCL-2 were more effective in treating prostate cancer NE than AR-positive prostate cancer (67). Also identified is MYCL as a novel driver of prostate cancer NE phenotype (67) which too was expressed in ACRJ-PC28 at RNA and protein levels. Fourth, AMACR is only expressed in prostate cancer NE cells, and this gene was expressed in ACRJ-PC28 at RNA and protein levels. Altogether, ACRJ-PC28 is more aligned with a prostate cancer NE phenotype and using PCA, the clustering of ACRJ-PC28 cells to prostate cancer versus normal prostate supports this claim. In addition, five prostate cancer–associated genes (*EGF*, *PIK3R1*, *ATM*, *BRCA1,* and *BRCA2*) with apparent damaging mutations, one of which is a rare mutation in ATM, a possible therapeutic target, further support this claim. Also of note is that numerous variants were detected in ACRJ-PC28 that are predicted to be deleterious.

Still, the cell line is atypical in expressing both basal (CK5 and p63) and luminal (CK18) markers and lacking the expression of chromogranin A, a typical NE biomarker, but expressing SYP and ENO2. The presence of ACRJ-PC28 further highlights the value of this cell line as currently, there is need for more research demystifying the transitional processes in the development of prostate cancer. Also, given that MAA are more likely to develop prostate cancer and die from the disease, having a transitional prostate cell line presents a valuable tool in understanding the dynamics of prostate cancer development and underlying factors that increases such risks.

Given that ACRJ-PC28 was from the TRNB of a patient diagnosed with late-stage prostate cancer adenocarcinoma, the initial hypothesis was that ACRJ-PC28 would be naturally immortal. However, prior to immortalization, these cells senesced at passage 6. Furthermore, none of the earlier 27 prostate cancer explants survived beyond passage 7. This indicated that these cells were susceptible to culture stress and/or replicative senescence under *in vitro* culture conditions. One possibility could be that our culture conditions induced cellular stress that predictably led to increased expression of p16/p14ARF. Alternatively, or additionally, our *in vitro* environment did not support expression of telomerase. To minimize alterations that could select if we only eliminated senescence pathways or ectopically expressed hTERT, we hypothesized that a two-hit immortalization through hTERT upregulation and *CDKN2A* ablation as described in Zhao and colleagues (20) would be most effective for rapid immortalization of cells that otherwise senesce. In this regard, our prior attempts at immortalization through p53 (dominant negative R175H mutant)-pcw107-V5 and hTERT upregulation, which does not target p16, proved ineffective and as such were not attempted on ACRJ-PC28.

Immunoblotting results expectedly indicated that ACRJ-PC28 lacked expression from the *CDKN2A* locus, as cells were negative for both p16 and p14^ARF^, which was consistent with the WGS results that indicated the possible heterozygous deletion at region chr9:21970801-22006400, when using CNVnator. The protein expression of both p53 and pRB tumor suppressors appeared normal, which also seems to correlate with WGS data that identified no exonic mutations, but intronic variants with no association to protein function. Recent research (68) highlighted the challenges in treating AR active adenocarcinomas that were associated with a combined loss of p53 and pRB. The tumors were found to be nonresponsive to AR antagonists. On the contrary, ACRJ-PC28 presents the reverse profile, the absence of AR and the presence of p53 and pRB.

The *Cannabis sativa extract* THC_EX3 that was three times more potent in reducing the viability of ACRJ-PC28 compared with PC-3 not only confirms the need for diverse cell lines but also can be a novel lead to be further explored against Erβ. Although preliminary, this further reiterates the suggestion that MAA can potentially derive greater benefits from treatment options for prostate cancer if more research accommodates the use of cell lines derived from MAA or through greater recruitment of MAA for clinical trials (69). Also highlighted was the IC_50_ value (12 μg/mL) of THC_EX3, which is significantly lower than widely used prostate cancer drug docetaxel (IC_50_ of 483 μg/mL) in PC-3 cells (70). Further purification of THC_EX3 could potentially provide a new targeted compound for treating prostate cancer with possibility for greater treatment derived benefits for MAA with the disease.

## Supplementary Material

Supplemental Figure SF1: STR profiling for ACRJ-PC28 cells. STR profile of ACRJ-PC28 generated by IDEXX using PC indicates the novelty of the cell line (A) as the cell line senctic profile was compared to the profiles of other cell lines in the DSMZ STR database and did not match anv other reported profiles here: (B) that the cclls were purely of humar origin and (C) the absence of mycoplasma intection

Supplemental Figure SF2: IHC staining on original patient tissue from which ACRJ-PC28 was derived. Tissue sample was obtained from a transrectal needle biopsy of the prostate gland of a patient with PSA

Supplemental Figure SF3: IHC staining on ACRJ-PC28 cells compared to positive controls. IHC staining on ACRJ-PC28 cells compared to positive controls.

Supplemental Figure SF4: The cytotoxic effect of a Cannabis extract, THC_EX3 on the viability of ACRJ-CP28 and PC3 cell line. Cell viability was determined using the MTS assay and OD readings were taken a 409nm. Plots were generated in Microsoft Excel and each data point represents the average of three replicates. Experiments shown are representative of two independent experiments.

Supplemental table ST1: Sample collection and culture conditions In these prior attempts, culture conditions such as media, extra-cellular matrices and culture flasks were varied to determine the optimal culture conditions for generation of prostate cancer explants.

Supplemental Table ST2: Supplemental Table 2: A search in the database of global cell repositories ATCC and ECACC revealed that approximately 40% of available prostate derived lines were immortalized via an exogenous source.

Supplementary Table ST3 All Exonic Variants which are present in ACRJ-PC28. SIFT, Polyphen2, LRT, MutationTaster, MutationAssessor, and CADD were used to predict variant effect. Pop_Freq_max is the maximum population frequency of the variant in 1000Genome Project (Aug 2015), Exome Sequence Project (ESP6500) and Genome Aggregation Database (gnomAD). Total_Damaging represents number of times the variant was predicted as damaging by the 5 methods used. There are 605 variants that are predicted as damaging for 5 methods (green) while 296 variants are predicted as damaging for 4 out of 5 methods (orange-brown), among them is the variant in ATM (rs147934285) (dk brown). 3). Additionally, 539 variants are predicted as damaging for 3 out of 5 methods (blue-purple). The data generated and/or analyzed during the current study are available from the corresponding author on reasonable request.

Supplementary Table ST4 Data from RNA-Seq analysis. Noteworthy were the gene ontologies with a high proportion of expressed genes, such as the nuclear compartment (28%), extracellular exosome compartment (15%), regulation of transcription (8%), apoptotic process (3%) and oxidation-reduction process (3%).

Supplementary Table ST5 Prostate cancer with neuroendocrine phenotype over-expresses MKI67, CD44, MYC, KLF4, MUC1, ENO2, POU5F1, EZH2, and SOX2. AR and KLK3 expression lost in neuroendocrine phenotype.
