# Supplementary material for: Expanding the Prostate Cancer Cell Line Repertoire with ACRJ-PC28, an AR-negative Neuroendocrine Cell Line Derived From an African-Caribbean Patient
Source: Cancer Res Commun. 2022 Nov 7;2(11):1355–71. doi: 10.1158/2767-9764.CRC-22-0245 (PMC9836004; doi:10.1158/2767-9764.CRC-22-0245)
Supplement: Supplemental Figure SF1: STR profiling for ACRJ-PC28 cells. — STR profile of ACRJ-PC28 generated by IDEXX using PC indicates the novelty of the cell line (A) as the cell line senctic profile was compared to the profiles of other cell lines in the DSMZ STR database and did not match anv other reported profiles here: (B) that the cclls were purely of humar origin and (C) the absence of mycoplasma intection [file crc-22-0245-s01.pptx]

## Slide 1
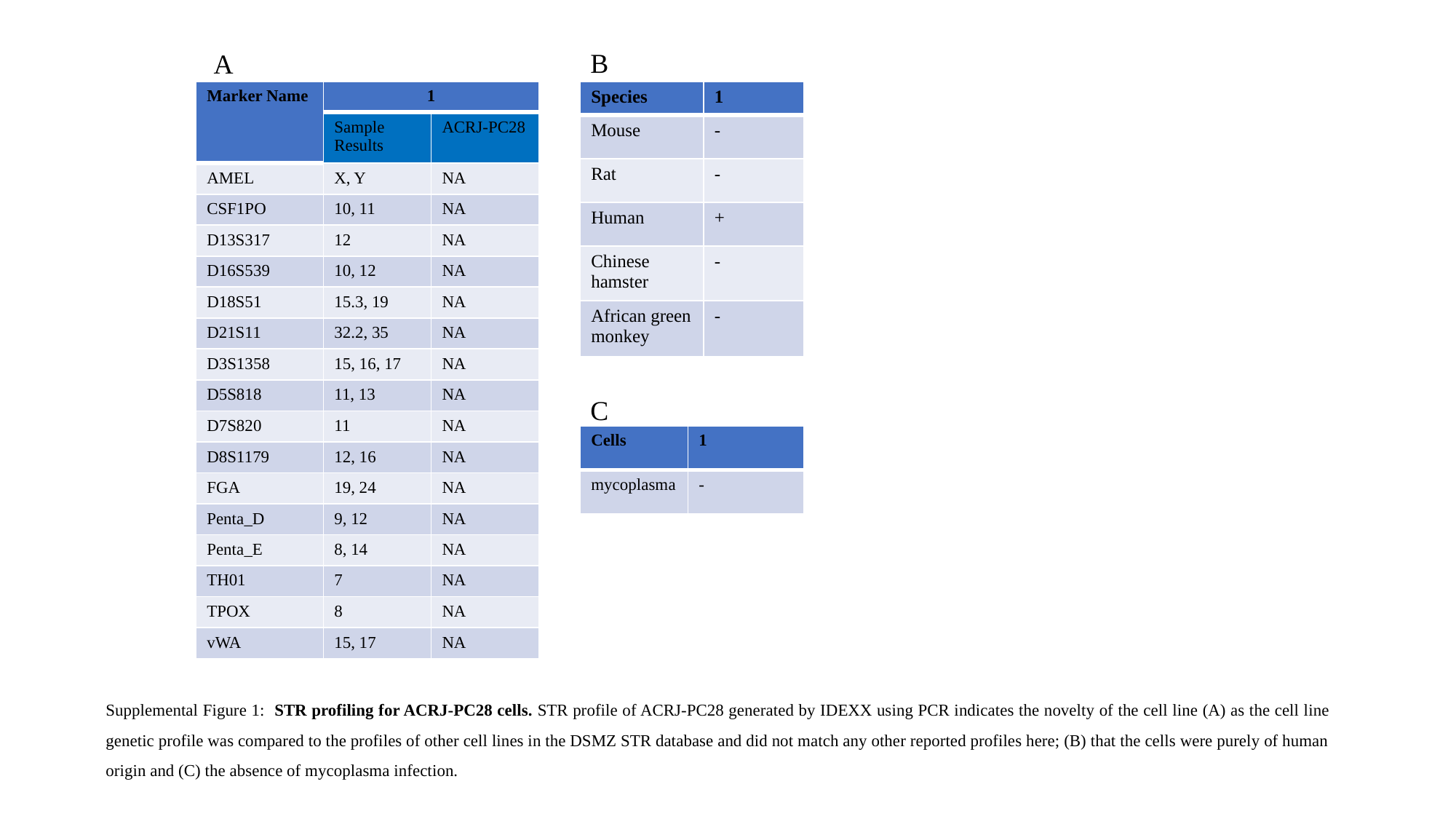

B
A
| Marker Name | 1 | |
| --- | --- | --- |
| | Sample Results | ACRJ-PC28 |
| AMEL | X, Y | NA |
| CSF1PO | 10, 11 | NA |
| D13S317 | 12 | NA |
| D16S539 | 10, 12 | NA |
| D18S51 | 15.3, 19 | NA |
| D21S11 | 32.2, 35 | NA |
| D3S1358 | 15, 16, 17 | NA |
| D5S818 | 11, 13 | NA |
| D7S820 | 11 | NA |
| D8S1179 | 12, 16 | NA |
| FGA | 19, 24 | NA |
| Penta\_D | 9, 12 | NA |
| Penta\_E | 8, 14 | NA |
| TH01 | 7 | NA |
| TPOX | 8 | NA |
| vWA | 15, 17 | NA |
| Species | 1 |
| --- | --- |
| Mouse | - |
| Rat | - |
| Human | + |
| Chinese hamster | - |
| African green monkey | - |
C
| Cells | 1 |
| --- | --- |
| mycoplasma | - |
Supplemental Figure 1: STR profiling for ACRJ-PC28 cells. STR profile of ACRJ-PC28 generated by IDEXX using PCR indicates the novelty of the cell line (A) as the cell line genetic profile was compared to the profiles of other cell lines in the DSMZ STR database and did not match any other reported profiles here; (B) that the cells were purely of human origin and (C) the absence of mycoplasma infection.
