# Supplementary material for: Expanding the Prostate Cancer Cell Line Repertoire with ACRJ-PC28, an AR-negative Neuroendocrine Cell Line Derived From an African-Caribbean Patient
Source: Cancer Res Commun. 2022 Nov 7;2(11):1355–71. doi: 10.1158/2767-9764.CRC-22-0245 (PMC9836004; doi:10.1158/2767-9764.CRC-22-0245)
Supplement: Supplemental Figure SF2: IHC staining on original patient tissue from which ACRJ-PC28 was derived. — Tissue sample was obtained from a transrectal needle biopsy of the prostate gland of a patient with PSA [file crc-22-0245-s02.pptx]

## Slide 1
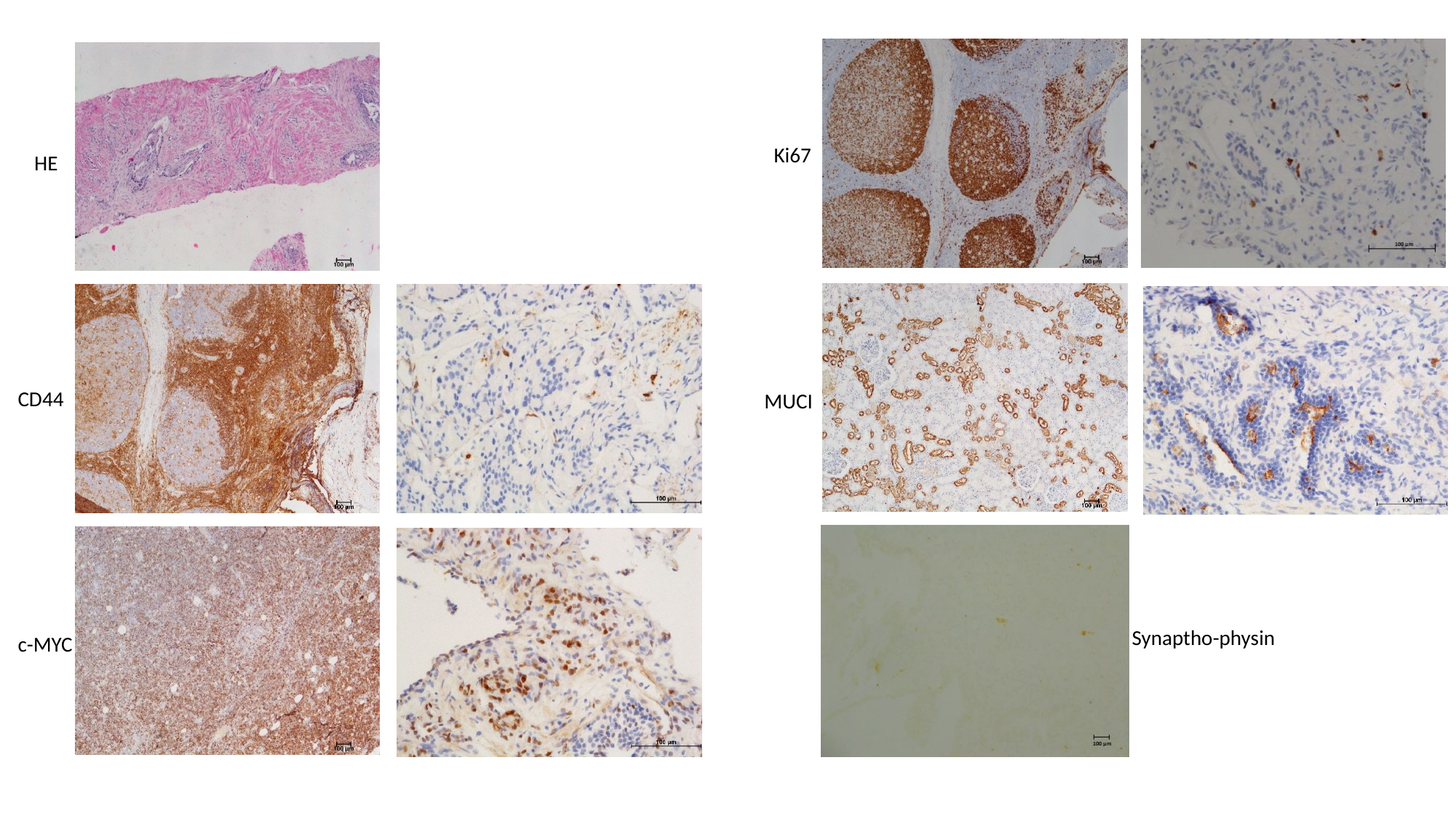

Ki67
HE
CD44
MUCI
Synaptho-physin
c-MYC

## Slide 2
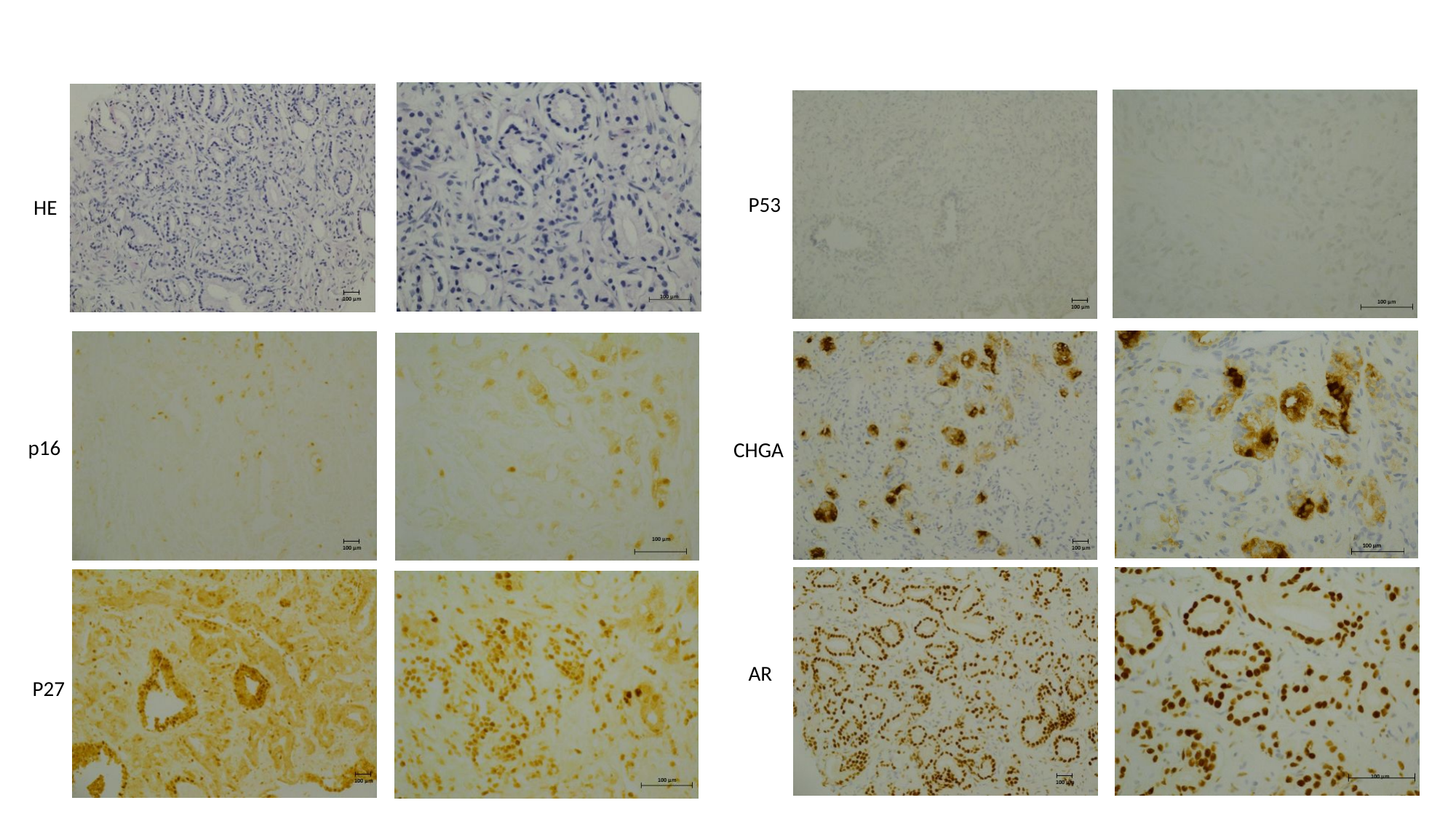

P53
HE
p16
CHGA
100 µm
AR
P27

## Slide 3
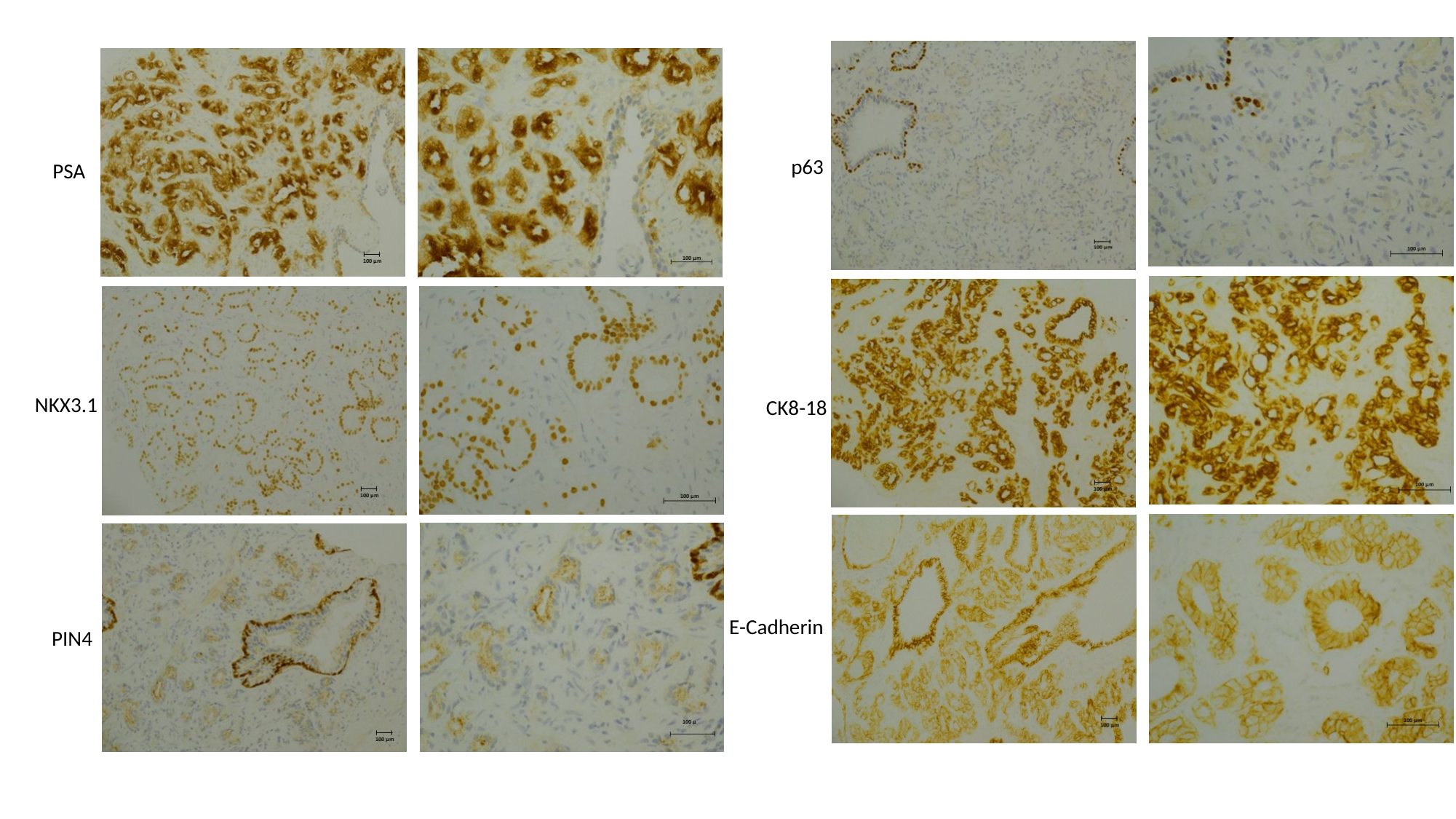

p63
PSA
NKX3.1
CK8-18
E-Cadherin
PIN4

## Slide 4
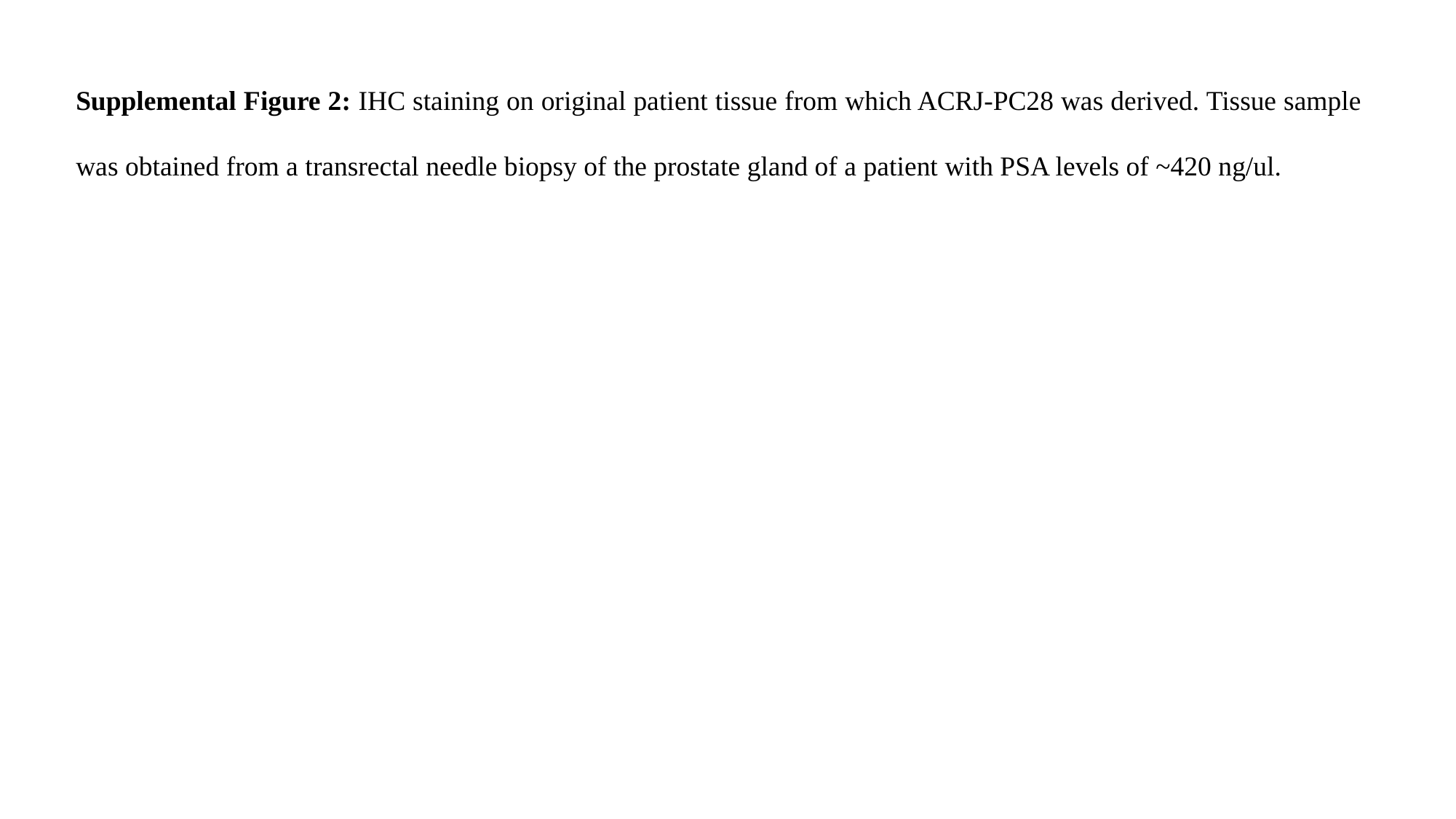

Supplemental Figure 2: IHC staining on original patient tissue from which ACRJ-PC28 was derived. Tissue sample was obtained from a transrectal needle biopsy of the prostate gland of a patient with PSA levels of ~420 ng/ul.
