# Supplementary material for: Expanding the Prostate Cancer Cell Line Repertoire with ACRJ-PC28, an AR-negative Neuroendocrine Cell Line Derived From an African-Caribbean Patient
Source: Cancer Res Commun. 2022 Nov 7;2(11):1355–71. doi: 10.1158/2767-9764.CRC-22-0245 (PMC9836004; doi:10.1158/2767-9764.CRC-22-0245)
Supplement: Supplemental Figure SF3: IHC staining on ACRJ-PC28 cells compared to positive controls. — IHC staining on ACRJ-PC28 cells compared to positive controls. [file crc-22-0245-s03.pptx]

## Slide 1
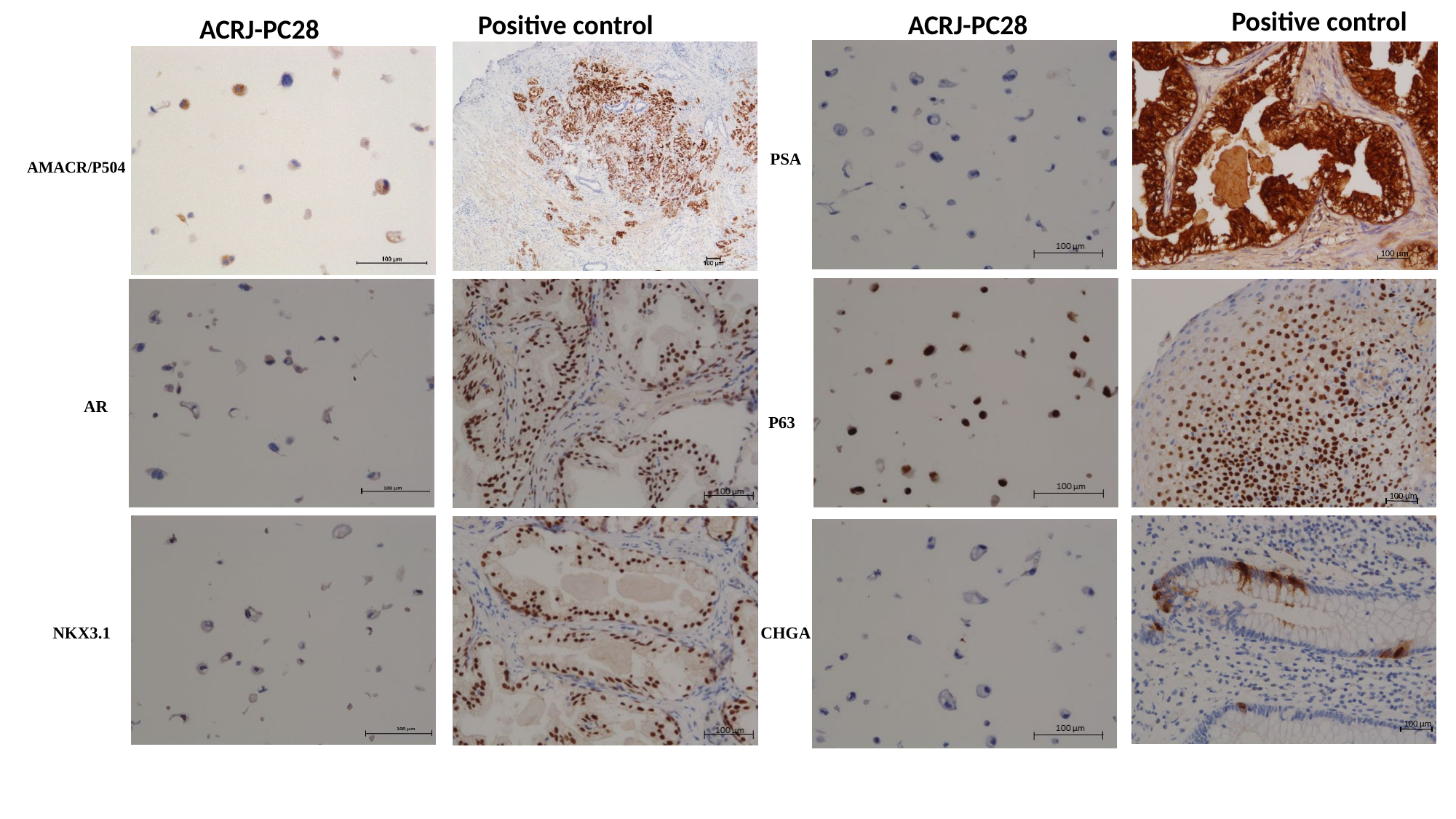

Positive control
Positive control
ACRJ-PC28
ACRJ-PC28
PSA
AMACR/P504
100 µm
AR
P63
100 µm
NKX3.1
CHGA
100 µm

## Slide 2
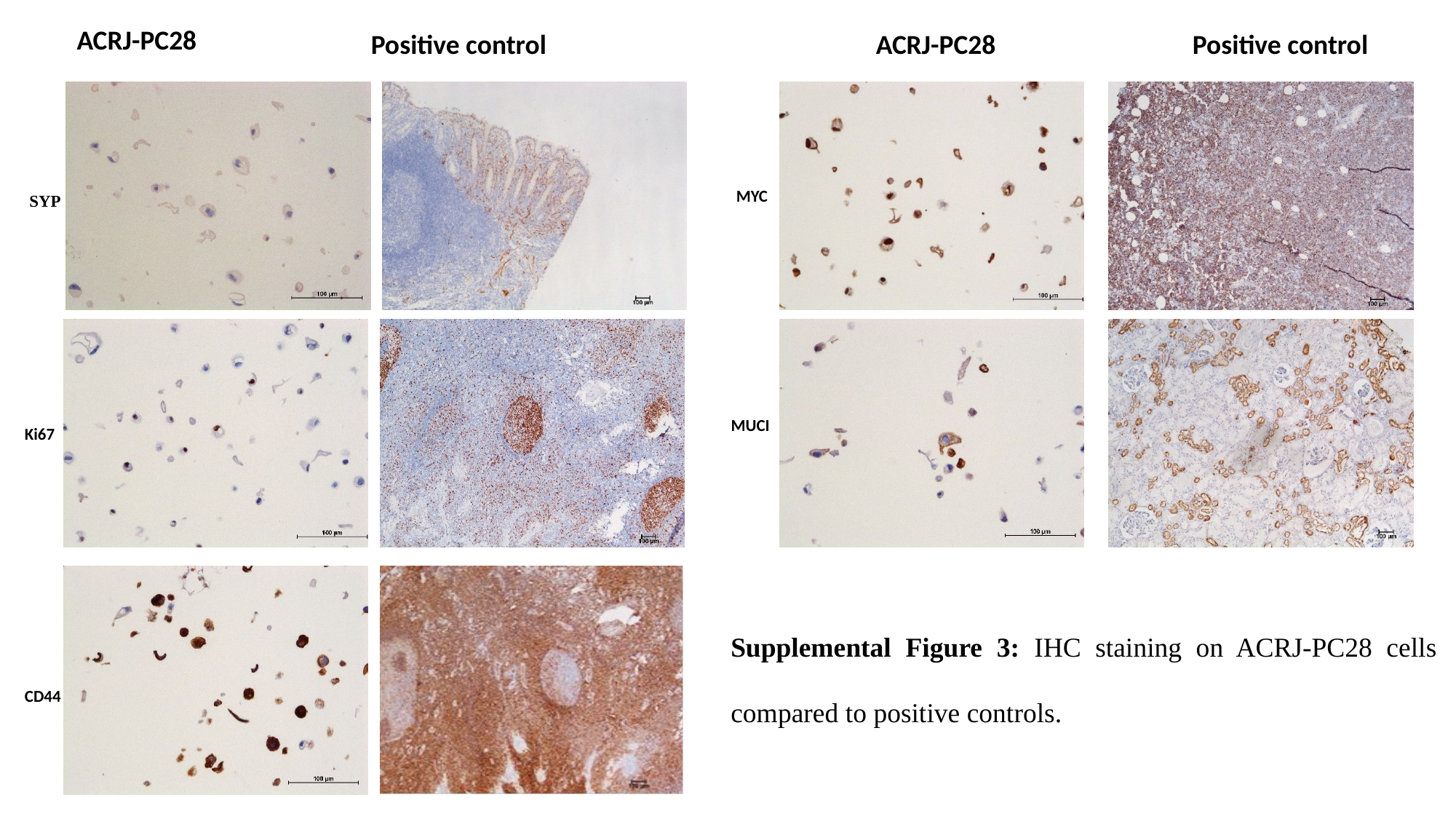

ACRJ-PC28
Positive control
Positive control
ACRJ-PC28
MYC
SYP
MUCI
Ki67
Supplemental Figure 3: IHC staining on ACRJ-PC28 cells compared to positive controls.
CD44
