# Supplementary material for: Expanding the Prostate Cancer Cell Line Repertoire with ACRJ-PC28, an AR-negative Neuroendocrine Cell Line Derived From an African-Caribbean Patient
Source: Cancer Res Commun. 2022 Nov 7;2(11):1355–71. doi: 10.1158/2767-9764.CRC-22-0245 (PMC9836004; doi:10.1158/2767-9764.CRC-22-0245)
Supplement: Supplemental Figure SF4: The cytotoxic effect of a Cannabis extract, THC_EX3 on the viability of ACRJ-CP28 and PC3 cell line. — Cell viability was determined using the MTS assay and OD readings were taken a 409nm. Plots were generated in Microsoft Excel and each data point represents the average of three replicates. Experiments shown are representative of two independent experiments. [file crc-22-0245-s04.pptx]

## Slide 1
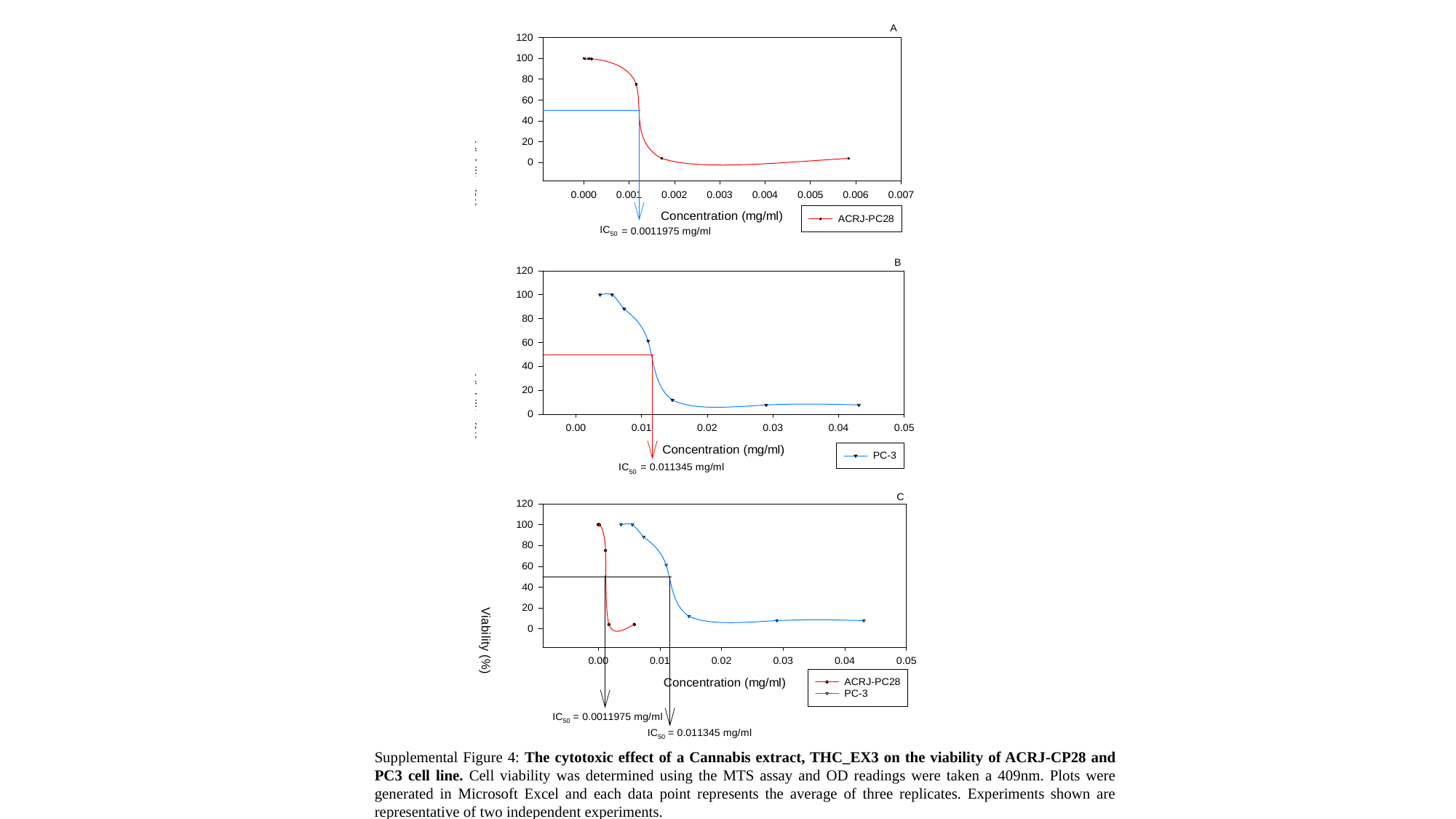

Supplemental Figure 4: The cytotoxic effect of a Cannabis extract, THC_EX3 on the viability of ACRJ-CP28 and PC3 cell line. Cell viability was determined using the MTS assay and OD readings were taken a 409nm. Plots were generated in Microsoft Excel and each data point represents the average of three replicates. Experiments shown are representative of two independent experiments.
