# Supplementary material for: Expanding the Prostate Cancer Cell Line Repertoire with ACRJ-PC28, an AR-negative Neuroendocrine Cell Line Derived From an African-Caribbean Patient
Source: Cancer Res Commun. 2022 Nov 7;2(11):1355–71. doi: 10.1158/2767-9764.CRC-22-0245 (PMC9836004; doi:10.1158/2767-9764.CRC-22-0245)
Supplement: Supplemental table ST1: Sample collection and culture conditions — In these prior attempts, culture conditions such as media, extra-cellular matrices and culture flasks were varied to determine the optimal culture conditions for generation of prostate cancer explants. [file crc-22-0245-s05.docx]

| **Sample** | **Culture surface** | **Compartment** | | **Extra Cellular- Matrix** | | **Growth Medium** | |
| --- | --- | --- | --- | --- | --- | --- | --- |
| **ACRJ-PC1** | 6-well plate was utilized for cell culture | Well A | | Matrigel in KSFM (50/50) | | Keratinocyte Growth Medium | |
|  |  | Well B | | Matrigel in F:12K (50/50) | | F:12K with 10% FBS | |
|  |  | Well C | | Matrigel in RPMI (50/50) | | RPMI with 10% FBS | |
|  |  | Well D | | Matrigel in DMEM (50/50) | | DMEM with 10% FBS | |
| **ACRJ-PC2** | 2-Well Chamber Plates were used | Chamber 1 | | Matrigel in KSFM (50/50) | | Keratinocyte Growth Medium | |
|  |  | Chamber 2 | | Matrigel in F:12K (50/50) | | F:12K with 10% FBS | |
|  |  | Chamber 3 | | Matrigel in RPMI (50/50) | | DMEM with 10% FBS | |
|  |  | Chamber 4 | | Matrigel in DMEM 50/50) | | RPMI with 10% FBS | |
| **ACRJ-PC3** | 2-Well Chamber Plates were used | Chamber 1 | | Matrigel in KSFM (50/50) | | Keratinocyte Growth Medium | |
|  |  | Chamber 2 | | Matrigel in F:12K (50/50) | | F:12K with 10% FBS | |
|  |  | Chamber 3 | | Matrigel in RPMI (50/50) | | RPMI with 10% FBS | |
|  |  | Chamber 4 | | Matrigel in DMEM (50/50) | | RPMI with 10% FBS | |
| **ACRJ-PC4** | 2-Well Chamber Slide | | RPMI in Matrigel (50/50) | | RPMI with 10% FBS, 10% NBCS | |  |
| **ACRJ-PC5** | 2-Well Chamber Slide | | KGM in Matrigel (50/50) | | KGM | |  |
|  |  |  | DMEM in Matrigel (50/50) | | DMEM with 10% FBS, 10% NBCS | |  |
| **ACRJ-PC6** | 2-Well Chamber Slide | | F12K in Matrigel (50/50) | | F12K with 10% FBS, 10% NBCS | |  |
| **ACRJ-PC7** | 2-Well Chamber Slide | | 0.1-0.2% gelatin | | DMEM with 10% FBS, 10% NBCS | |  |
|  |  |  | 0.1-0.2% gelatin | | F12K with 10% FBS, 10% NBCS | |  |
| **ACRJ-PC8** | 2-Well Chamber Slide | | 0.1-0.2% gelatin | | DMEM:F12K (50/50) with 10% FBS, 10% NBCS | |  |
|  |  |  | 0.1-0.2% gelatin | | F12K with 10% FBS, 10% NBCS | |  |
| **ACRJ-PC9** | 2-Well Chamber Slide | | 0.1-0.2% gelatin | | DMEM: F12K (50/50) with 10% FBS, 10% NBCS with 1% penicillin/streptomycin and 0.1% Amphotericin. | |  |
|  |  | | No ECM | | DMEM: F12K (50/50) with 10% FBS, 10% NBCS with 1% penicillin/streptomycin and 0.1% Amphotericin | |  |
| **ACRJ-PC10** | 2-Well Chamber Slide | | 0.01% collagen | | F12:K supplemented with 10% FBS and 10% NBCS | |  |
|  |  | | 0.01% collagen | | DMEM supplemented with 10% FBS and 10% NBCS | |  |
|  |  | | 0.2 % gelatin | | F12K supplemented with 10% FBS and 10% NBCS | |  |
|  |  | | 0.2% gelatin | | DMEM supplemented with 10% FBS and 10% NBCS | |  |
| **ACRJ-PC11** |  | | 0.1% gelatin | | RPMI with 10% FBS and 10% NBCS | |  |
|  | 2-Well Chamber Slide | | 0.2% gelatin | | RPMI supplemented with 10% FBS and 10% NBCS | |  |
|  |  | | 0.1% gelatin | |  | |  |
|  |  | | 0.2% gelatin | | DMEM supplemented with 10% FBS and 10% | |  |
| **ACRJ-PC12** |  | | 0.2 % gelatin | | DMEM: F12K (50/50) supplemented with 10% NBCS and 10% FBS | |  |
|  | 2-Well Chamber plate | | 0.01 % collagen | | F12K with 10% FBS and 10% NBCS | |  |
| **ACRJ-PC13** |  | | ATCC 56x2 feeder monolayer | | DMEM: F12K (50/50) with 10%FBS and 10% NBCS | |  |
|  | 2-Well chamber plate | | ATCC 56x2 feeder monolayer | | KGM  DMEM with 10%FBS and 10% NBCS  KGM | |  |
|  |  | |  | |  | |  |
| **ACRJ-PC14** | 2-Well chamber plate | | ATCC 56x2 feeder monolayer | | DMEM:F12K (50/50) with 10% FBS and 10% NBCS  KGM | |  |
|  |  | |  | |  | |  |
| **ACRJ-PC15** | 2-Well chamber plate and  25 cm^2^ culture flask | | ATCC 56X2 feeder monolayer  ATCC 56X2 feeder monolayer | | DMEM:F12K (50/50) with 10% FBS and 10% NBCS  KGM | |  |
| **ACRJ-PC16** | 6-Well chamber plate | | ATCC 56X2 feeder monolayer | | DMEM:F12K (50/50) with 10% FBS and 10% NBCS  KGM | |  |
|  |  | | ATCC 56X2 feeder monolayer | | DMEM:F12K (50/50) with 10% FBS and 10% NBCS  KGM | |  |
| **ACRJ-PC17** | 6-Well chamber plate | | ATCC 56X2 feeder monolayer  0.01% Collagen type 1 | | DMEM:F12K (50/50) with 10% FBS and 10% NBCS  KGM | |  |
|  |  | | ATCC 56X2 feeder monolayer | | DMEM:F12K with 10% FBS and 10% NBCS  KGM | |  |
| **ACRJ-NP18**  **(Normal prostate tissue)** | 6-Well chamber plate | | 3T3 swiss albino irradiated feeder monolayer | | DMEM:F12K (50/50) with 20% FBS  KGM | |  |
| **ACRJ-PC19** | 6-Well chamber plate | | 3T3 swiss albino irradiated feeder monolayer | | DMEM:F12K (50/50) with 20% FBS  MKGM | |  |
| **ACRJ-PC20** | 6-Well chamber plate | | 3T3 swiss albino irradiated feeder monolayer | | DMEM:F12K (50/50) with 20% FBS  MKGM | |  |
| **ACRJ-PC21** | 6-Well chamber plate | | 3T3 swiss albino irradiated feeder monolayer  0.01% Collagen type 1 | | DMEM:F12K (50/50) with 20% FBS  MKGM | |  |
| **ACRJ-PC22** | 6-Well chamber plate | | 3T3 swiss albino irradiated feeder monolayer  0.01% Collagen type 1 | | DMEM:F12K (50/50) with 20% FBS  MKGM | |  |
| **ACRJ-PC23** | 6-Well chamber plate | | 0.01% Collagen type 1 | | DMEM:F12K (50/50) with 20% FBS  MKGM | |  |
| **ACRJ-PC24** | 6-Well chamber plate | | 0.01% Collagen type 1 | | DMEM:F12K (50/50) with 20% FBS  MKGM | |  |
| **ACRJ-PC25** | 6-Well chamber plate | | 0.01% Collagen type 1 | | DMEM:F12K (50/50) with 20% FBS  MKGM | |  |
| **ACRJ-PC26** | 6-Well chamber plate | | 0.01% Collagen type 1 | | DMEM:F12K (50/50) with 20% FBS  MKGM | |  |
| **ACRJ-PC27** | 6-Well chamber plate | | 3T3 Swiss iJ2 feeder cells  0.01% Collagen type 1 | | DMEM:F12K (50/50) with 20% FBS  MKGM | |  |
| **ACRJ-PC28** | 6-Well chamber plate | | 3T3 Swiss iJ2 feeder cells  0.01% Collagen type 1 | | DMEM:F12K (50/50) with 20% FBS  MKGM | |  |

**Supplemental table 1:** Sample collection and culture conditions. ACRJ-PC28 was generated after 27 prior attempts. In these prior attempts, culture conditions such as media, extra-cellular matrices and culture flasks were varied to determine the optimal culture conditions for generation of prostate cancer explants.

**Keratinocyte Growth Medium (KGM)**:

Keratinocyte serum-free medium, Epidermal growth factor (EGF; 5 ng/mL), Bovine pituitary extract (BPE; 50 m g/mL)

**Max Keratinocyte Growth Medium (MKGM)**

Keratinocyte serum-free medium, Epidermal growth factor (EGF; 5 ng/mL), Bovine pituitary extract (BPE; 50 µg/mL), Hydrocortisone (100 ng/ml), Insulin (5µg/ml), Apo-Transferin (5µg/ml), Recombinant Tumor Growth Factor- Alpha (0.5 ng/ml)
