# Supplementary material for: Expanding the Prostate Cancer Cell Line Repertoire with ACRJ-PC28, an AR-negative Neuroendocrine Cell Line Derived From an African-Caribbean Patient
Source: Cancer Res Commun. 2022 Nov 7;2(11):1355–71. doi: 10.1158/2767-9764.CRC-22-0245 (PMC9836004; doi:10.1158/2767-9764.CRC-22-0245)
Supplement: Supplemental Table ST2: — Supplemental Table 2: A search in the database of global cell repositories ATCC and ECACC revealed that approximately 40% of available prostate derived lines were immortalized via an exogenous source. [file crc-22-0245-s06.docx]

|  | **Cell line** | **Ethnicity** | **Tumor site** | **Immortalization method** |
| --- | --- | --- | --- | --- |
| **ATCC** |  |  |  |  |
|  | 22Rv1(Sramkoski et al., 1999) | White | Xenograft derived following castration of androgen-dependent parental line CWR22 xenograft | - |
|  | C4(Pfitzenmaier et al., 2003) | White | LNCaP derived | - |
|  | C4-2 (Pfitzenmaier et al., 2003) | White | LNCaP derived | - |
|  | C4-2b(Pfitzenmaier et al., 2003) | White | LNCaP-derived C4-2 cells | - |
|  | DU-145(Stone et al., 1978) | White | Brain metastasis | Spontaneous |
|  | LASCPC-01(Lee et al., 2016) | White | Benign prostate tissue | N-Myc driver with AKT-1 activation |
|  | LNCaP(Horoszewicz et al., 1983) | White | Lymph Node metastasis | Spontaneous |
|  | MDA PCa 2b(Navone et al., 1997) | Black | Bone Metastasis from a black male with androgen-independent adenocarcinoma of the prostate | Spontaneous |
|  | PWR-1E(Webber et al., 1996) | White | Normal prostate | Adenovirus 12-SV40 hybrid virus (Ad12-SV40) |
|  | RPWE-1(Bello et al., 1997) | White | Normal adult human prostate | human papilloma virus 18 (HPV-18) |
|  | RPWE-2(Bello et al., 1997) | White | RWPE-2 cells were derived from RWPE-1 cells | Transformation with Ki-ras using the Kirsten murine sarcoma virus (Ki-MuSV) |
|  | RPWE-2-W99(Bello et al., 1997) | White | Derived from RWPE-2 cell line by cloning in soft agar to select cells that show high expression of Ki-ras | - |
|  | VCaP(Korenchuk et al., 2001) | White | Bone Metastasis | Spontaneous |
|  | WPE1-NB14(Bello et al., 1997) | White | WPE1-NB14 cells were derived from RWPE-1 cells (ATCC CRL-11609) after exposure to N-methyl-N-nitrosourea (MNU) | - |
|  | WPE1-NB26(Bello et al., 1997) | White | RWPE-1 derived after exposure to N-methyl-N-nitrosourea (MNU) | - |
| **ECACC** |  |  |  |  |
|  | BOB(Attard et al., 2009) | White | Spontaneously immortalized prostate cancer cell line established from a transrectal needle biospy | Spontaneous |
|  | P4E6(Maitland et al., 2001) | White | P4E6 is an Immortalised human prostate cell line derived from a biopsy of a well-differentiated early stage prostate cancer | HPV type E6 gene from PLXSN16E6 retroviral stock |
|  | PNTA1(Cussenot et al., 1991) | White | Human post pubertal prostate normal | SV40 |
|  | PNTA2(Berthon et al., 1995) | White | Normal adult prostatic epithelial cells by transfection with a plasmid containing SV40 genome with a defective replication origin | SV40 Large T antigen |
|  | Shmac 1  (Unpublished) | - | Shmac 1 is an immortalised human prostate cell line which was immortalised using human papillomavirus type E6 gene from PLXSN16E6 retroviral stock | HPV s type E6 gene from PLXSN16E6 retroviral stock |
|  | Shmac 4(Lang et al., 2006) | White | Shmac 4 is an immortalised human prostate cell line derived from a biopsy of a well-differentiated early stage prostate cancer (Gleason grade (1+2)) | human papillomavirus type E6 gene from PLXSN16E6 |
|  | Shmac 5(Lang et al., 2006) | White | Shmac 5 is an Immortalised human prostate cell line derived from a biopsy of a moderately well differentiated early stage prostate cancer (Gleason grade (3+3) | human papillomavirus type E6 gene from PLXSN16E6 |

**Supplemental table 2:** Human prostate-derived cell lines available at two major cell repositories ATCC and ECACC. A search in the database of global cell repositories ATCC and ECACC revealed that approximately 40% of available prostate derived lines were immortalized via an exogenous source.

**References**

Attard, G., Rizzo, S., Ledaki, I., Clark, J., Reid, A. H., Thompson, A., Khoo, V., de Bono, J. S., Cooper, C. S., & Hudson, D. L. (2009). A novel, spontaneously immortalized, human prostate cancer cell line, Bob, offers a unique model for pre-clinical prostate cancer studies. *Prostate*, *69*(14), 1507-1520. <https://doi.org/10.1002/pros.20997>

Bello, D., Webber, M. M., Kleinman, H. K., Wartinger, D. D., & Rhim, J. S. (1997). Androgen responsive adult human prostatic epithelial cell lines immortalized by human papillomavirus 18. *Carcinogenesis*, *18*(6), 1215-1223. <https://doi.org/10.1093/carcin/18.6.1215>

Berthon, P., Cussenot, O., Hopwood, L., Leduc, A., & Maitland, N. (1995). Functional expression of sv40 in normal human prostatic epithelial and fibroblastic cells - differentiation pattern of nontumorigenic cell-lines. *Int J Oncol*, *6*(2), 333-343. <https://doi.org/10.3892/ijo.6.2.333>

Cussenot, O., Berthon, P., Berger, R., Mowszowicz, I., Faille, A., Hojman, F., Teillac, P., Le Duc, A., & Calvo, F. (1991). Immortalization of human adult normal prostatic epithelial cells by liposomes containing large T-SV40 gene. *J Urol*, *146*(3), 881-886. <https://doi.org/10.1016/s0022-5347(17)37953-3>

Horoszewicz, J. S., Leong, S. S., Kawinski, E., Karr, J. P., Rosenthal, H., Chu, T. M., Mirand, E. A., & Murphy, G. P. (1983). LNCaP model of human prostatic carcinoma. *Cancer Res*, *43*(4), 1809-1818.

Korenchuk, S., Lehr, J. E., L, M. C., Lee, Y. G., Whitney, S., Vessella, R., Lin, D. L., & Pienta, K. J. (2001). VCaP, a cell-based model system of human prostate cancer. *In Vivo*, *15*(2), 163-168.

Lang, S. H., Smith, J., Hyde, C., Macintosh, C., Stower, M., & Maitland, N. J. (2006). Differentiation of prostate epithelial cell cultures by matrigel/ stromal cell glandular reconstruction. *In Vitro Cell Dev Biol Anim*, *42*(8-9), 273-280. <https://doi.org/10.1290/0511080.1>

Lee, J. K., Phillips, J. W., Smith, B. A., Park, J. W., Stoyanova, T., McCaffrey, E. F., Baertsch, R., Sokolov, A., Meyerowitz, J. G., Mathis, C., Cheng, D., Stuart, J. M., Shokat, K. M., Gustafson, W. C., Huang, J., & Witte, O. N. (2016). N-Myc Drives Neuroendocrine Prostate Cancer Initiated from Human Prostate Epithelial Cells. *Cancer Cell*, *29*(4), 536-547. <https://doi.org/10.1016/j.ccell.2016.03.001>

Maitland, N. J., Macintosh, C. A., Hall, J., Sharrard, M., Quinn, G., & Lang, S. (2001). In vitro models to study cellular differentiation and function in human prostate cancers. *Radiat Res*, *155*(1 Pt 2), 133-142. [https://doi.org/10.1667/0033-7587(2001)155[0133:ivmtsc]2.0.co;2](https://doi.org/10.1667/0033-7587(2001)155%5b0133:ivmtsc%5d2.0.co;2)

Navone, N. M., Olive, M., Ozen, M., Davis, R., Troncoso, P., Tu, S. M., Johnston, D., Pollack, A., Pathak, S., von Eschenbach, A. C., & Logothetis, C. J. (1997). Establishment of two human prostate cancer cell lines derived from a single bone metastasis. *Clin Cancer Res*, *3*(12 Pt 1), 2493-2500.

Pfitzenmaier, J., Quinn, J. E., Odman, A. M., Zhang, J., Keller, E. T., Vessella, R. L., & Corey, E. (2003). Characterization of C4-2 prostate cancer bone metastases and their response to castration. *J Bone Miner Res*, *18*(10), 1882-1888. <https://doi.org/10.1359/jbmr.2003.18.10.1882>

Sramkoski, R. M., Pretlow, T. G., 2nd, Giaconia, J. M., Pretlow, T. P., Schwartz, S., Sy, M. S., Marengo, S. R., Rhim, J. S., Zhang, D., & Jacobberger, J. W. (1999). A new human prostate carcinoma cell line, 22Rv1. *In Vitro Cell Dev Biol Anim*, *35*(7), 403-409. <https://doi.org/10.1007/s11626-999-0115-4>

Stone, K. R., Mickey, D. D., Wunderli, H., Mickey, G. H., & Paulson, D. F. (1978). Isolation of a human prostate carcinoma cell line (DU 145). *Int J Cancer*, *21*(3), 274-281. <https://doi.org/10.1002/ijc.2910210305>

Webber, M. M., Bello, D., Kleinman, H. K., Wartinger, D. D., Williams, D. E., & Rhim, J. S. (1996). Prostate specific antigen and androgen receptor induction and characterization of an immortalized adult human prostatic epithelial cell line. *Carcinogenesis*, *17*(8), 1641-1646. <https://doi.org/10.1093/carcin/17.8.1641>
