# Supplementary material for: Expanding the Prostate Cancer Cell Line Repertoire with ACRJ-PC28, an AR-negative Neuroendocrine Cell Line Derived From an African-Caribbean Patient
Source: Cancer Res Commun. 2022 Nov 7;2(11):1355–71. doi: 10.1158/2767-9764.CRC-22-0245 (PMC9836004; doi:10.1158/2767-9764.CRC-22-0245)
Supplement: Supplementary Table ST5 — Prostate cancer with neuroendocrine phenotype over-expresses MKI67, CD44, MYC, KLF4, MUC1, ENO2, POU5F1, EZH2, and SOX2. AR and KLK3 expression lost in neuroendocrine phenotype. [file crc-22-0245-s09.docx]

| **Gene Symbol** | **Gene Name** | **Chromosome Location** | **TPM value** |
| --- | --- | --- | --- |
| MKI67*^ | Marker of proliferation Ki-67 | chr10: 128096659- 128126385 | 377.00 |
| CD44* | CD44 stem cell associated marker | chr11: 35138870- 35232402 | 329.99 |
| MYC* | MYC proto-oncogene, bHLH transcription factor | chr1: 127736084- 127741434 | 97.73 |
| KLF4*^ | Kruppel like factor 4 | chr9: 107484852- 107490482 | 25.53 |
| MUC1*^ | Mucin 1 | chr1: 155185824- 155192916 | 17.86 |
| ENO2* | Enolase 2 | chr1: 155185824- 155192916 | 15.47 |
| PSCA | Prostate stem cell antigen | chr8: 142670308- 142682724 | 13.13 |
| POU5F1 (OCT4)* ^ | POU class 5 homeobox 1 | chr6: 31164337- 31180731 | 9.84 |
| EZH2* | Enhancer of zeste 2 polycomb repressive complex 2 subunit | chr7: 148807383- 148884321 | 6.92 |
| AMACR | Alpha-methylacyl-CoA racemase | chr5: 33986178- 34008108 | 6.15 |
| COL1A1 | Collagen type I alpha 1 chain | chr17: 50183289- 50201632 | 5.38 |
| TPX2 | TPX2, microtubule nucleation factor | chr20: 31739271- 31801805 | 3.58 |
| BGN | Biglycan | chrX: 153494939- 153509554 | 1.84 |
| SOX2*^ | SRY-box 2 | chr3: 181711924- 181714436 | 1.43 |
| ACP3 (PAP, ACPP) | Acid phosphatase, prostate | chr3: 132317367- 132368298 | 0.22 |
| KLK3 (PSA) | Kallikrein related peptidase 3 | chr19: 50854915 - 50860764 | N.D. |
| AR | Androgen receptor | chrX: 67544032- 67730619 | N.D. |

Supplemental Table 5: Prostate cancer with neuroendocrine phenotype over-expresses MKI67, CD44, MYC, KLF4, MUC1, ENO2, POU5F1, EZH2, and SOX2. AR and KLK3 expression lost in neuroendocrine phenotype.

*Prostate cancer with neuroendocrine phenotype over-expresses MKI67, CD44, MYC, KLF4, MUC1, ENO2, POU5F1, EZH2, and SOX2. AR and KLK3 expression lost in neuroendocrine phenotype.

^Not detectable or 3 to 72-fold lower expression in prostate cancer cell lines MDA-PCa-2b, RC77T/E, VCaP, LNCaP and PC3. PC3 cells considered to display neuroendocrine phenotype (see **Figure 4**)

Chr = Chromosome

N.D. = Not detectable
